# Development and validation of next-generation sequencing-based clinical test for triazole resistance prediction in *Aspergillus fumigatus*

**DOI:** 10.1128/jcm.00291-25

**Published:** 2025-07-01

**Authors:** J. R. Caldera, Ashley Dayo, Nathan Wiederhold, Shangxin Yang

**Affiliations:** 1 Department of Pathology and Laboratory Medicine, David Geffen School of Medicine, UCLA Health 155697, Los Angeles, California, USA; 2 Department of Pathology and Laboratory Medicine, University of Texas Health Science Center at San Antonio 14742, San Antonio, Texas, USA; University of Utah, Salt Lake City, Utah, USA

**Keywords:** *Aspergillus fumigatus*, azole resistance, whole-genome sequencing, genotyping, antifungal resistance, next-generation sequencing, cyp51A mutations, antifungal susceptibility testing

## Abstract

The rising rates of triazole drug resistance in *Aspergillus fumigatus* have placed greater reliance on antifungal susceptibility testing (AFST) to guide therapeutic management, particularly in medically complicated patients. Current methods, however, utilize conventional phenotypic assays that may pose significant challenges in performance, result interpretation, and time to reporting. Herein, we developed and validated a next-generation sequencing-based clinical test to predict the AFST phenotype of *A. fumigatus* against voriconazole, posaconazole, isavuconazole, and itraconazole using the *cyp51A* genotype as a marker for susceptibility. We sequenced 109 isolates comprising reference and clinical samples from the University of California, Los Angeles, Clinical Microbiology Lab, University of Texas Health Science Center at San Antonio Fungus Testing Laboratory, and the Centers for Disease Control and Prevention and Food and Drug Administration Antimicrobial Resistance Isolate Bank. Additionally, we integrated data from 14 previously published studies to produce comprehensive and robust interpretive criteria for the assay. Despite the complex association between *cyp51A* mutations and drug resistance, our triazole resistance assay produced a remarkable negative percent agreement (specificity) of ≥95%, thus offering presumptive, yet clinically actionable identification of triazole-wild-type isolates. Clinically, this allows for the rapid discrimination between acquired microbiological resistance in *A. fumigatus* and observed clinical resistance due to patients’ declining health to help guide the most effective therapeutic management.

**IMPORTANCE:**

The rising rates of antifungal resistance have been expressed by many as “the silent pandemic,” profoundly reshaping the landscape of fungal disease management. Innovations in clinical mycology, however, have remained limited, particularly in comparison to the significant advances seen in the greater field of microbiology. Here, we sought to capitalize upon the expanding utility of next-generation sequencing to address a gap in clinical mycology diagnostics and antifungal susceptibility testing. We developed a whole-genome sequencing protocol to evaluate *Aspergillus fumigatus cyp51A* genotype to predict phenotypic susceptibility to triazole drugs. Our triazole resistance assay offers clinically actionable identification of triazole-wild-type isolates of *A. fumigatus* in a much more expeditious timeline than traditional phenotypic susceptibility testing.

## INTRODUCTION

*Aspergillus fumigatus* is a ubiquitous saprobic fungus that frequently causes respiratory infections in patients with predisposing conditions, such as chronic obstructive pulmonary disease and hematopoietic stem cell and solid organ transplant recipients, respectively (1). Worldwide estimates have found over 250,000 annual cases of invasive aspergillosis alone, with mortality rates ranging from 30% to 80% (2). The global burden of *A. fumigatus* infections is further worsened by the rising incidence of resistance to triazole antifungals, the first-line drugs for the treatment and management of *Aspergillus* infections (3–6).

Clinically available triazole drugs, which include voriconazole, posaconazole, isavuconazole, and itraconazole, primarily target Cyp51A/B cytochrome P450 14-α-sterol demethylase enzyme to inhibit the synthesis of ergosterol, a key component of fungal cell wall (7). Not surprisingly, resistance to triazole drugs has been associated with a significantly higher mortality rate from 50% to 100% (4, 8–11). While the true clinical prevalence of triazole resistance in *A. fumigatus* is difficult to ascertain due to its heterogeneity in different populations, the ARTEMIS Global Surveillance Study that included 497 isolates from 62 medical centers estimated that 5.8% of clinical samples have triazole minimum inhibitory concentrations (MICs) above established epidemiological cut-off values (ECVs), suggesting acquired antifungal resistance in these strains (3, 12). More alarmingly, a larger global survey from 2017 to 2021 consisting of 731 *A*. *fumigatus* isolates has indicated that rates of non-wild-type azole phenotype may be as high as 10.8% (13).

Correspondingly, understanding the mechanisms of triazole resistance has been the subject of significant research since they were first described in the late 1990s (3). Early studies have associated various acquisitions of resistance to either prior medical triazole exposure or agricultural triazole use. Specifically, resistance mechanisms in previously triazole-treated patients typically include non-synonymous point mutations within the *cyp51A* coding sequence (CDS), whereas tandem-repeat mutations in the *cyp51A* promoter region have been postulated to originate from environmental triazole exposure, given their discovery in triazole-naïve individuals (3). In a recent survey of *A. fumigatus* isolates in the U.S.A., 26% (46 out of 179) were found to have triazole MICs above established ECVs, of which 96% (44 out of 46) included mutations in the *cyp51A* gene (i.e., *cyp51A*-dependent resistance) (14). It is worth noting, however, that worldwide, variably 15%–50% of phenotypically resistant *A. fumigatus* isolates have been reported to have wild-type *cyp51A* genotype, highlighting the presence of *cyp51A*-independent mechanisms of resistance, such as mutations in efflux pumps and in the HMG-CoA enzyme (encoded by *hmg1*), which catalyzes the first committed step in ergosterol biosynthesis (3, 7, 15).

The changing landscape of antifungal resistance, particularly coupled with a growing population at risk of fungal infections, has complicated the clinical management of aspergillosis with a greater reliance on antifungal susceptibility testing (AFST). Currently, the gold standard for AFST is a culture-based phenotypic assay to determine the MICs of select antifungal drugs (16, 17). While published clinical standards are available for test performance and result interpretation, phenotypic assays pose a significant burden on routine clinical laboratories and are typically outsourced to specialty reference laboratories. Moreover, the fungal growth rate is relatively slow, prolonging the performance of AFST and further delaying clinical reporting. As such, treatment decisions are commonly made empirically based on published guidelines (5, 18). Additionally, difficulties in phenotypic AFST performance can introduce errors that may complicate test interpretation. In recognizing these challenges, we sought to capitalize upon the comprehensively characterized genomic landscape of *cyp51A*-mediated triazole resistance in *A. fumigatus* to develop and validate an in-house next-generation sequencing (NGS)-based triazole resistance prediction assay (“triazole resistance assay”) that can provide presumptive, yet clinically actionable results at a much more expeditious timeline.

## MATERIALS AND METHODS

### *Aspergillus fumigatus* isolates

A total of 109 *A*. *fumigatus* clinical and reference isolates were sequenced in this study sourced from the University of California, Los Angeles (UCLA) Clinical Microbiology Lab (*n* = 30), University of Texas Health Science Center at San Antonio (UT Health at San Antonio) Fungus Testing Laboratory (FTL) (*n* = 69) and the Centers for Disease Control and Prevention (CDC) and Food and Drug Administration Antimicrobial Resistance (AR) Isolate Bank (*n* = 10). A subset of 75 isolates was randomly collected and analyzed upon clinical request for AFST (“clinical subset”).

Species identification of the UCLA isolates was determined by morphology and/or matrix-assisted laser desorption/ionization time-of-flight mass spectrometry (MALDI-ToF MS), whereas identification of isolates from the UT Health at San Antonio FTL and CDC AR Bank was determined by their respective laboratories (19, 20). For isolates with poor alignment to the reference *A. fumigatus* sequence (indicating likelihood of other cryptic species within the complex, i.e., *Aspergillus lentulus*) (Fig. S1A, bottom panel), species identification using whole-genome sequencing (WGS) analysis was performed as previously described (21). Voriconazole, posaconazole, isavuconazole, and itraconazole AFSTs were performed by the UT Health at San Antonio FTL according to methods published in the Clinical and Laboratory Standards Institute (CLSI) M38 standard (17). Results for voriconazole and isavuconazole were interpreted according to current CLSI guidelines (22, 23). Results for posaconazole and itraconazole were categorized according to recently published ECVs (24). AFSTs for all isolates with non-susceptible MICs were repeated for reproducibility.

A comprehensive data set was created by supplementing the UCLA-sequenced sample set with previously published data genotypically and phenotypically profiling clinical and environmental *A. fumigatus* isolates (*n* = 14 international studies published in peer-reviewed journals reporting local clinical cases and/or large epidemiological surveys of *A. fumigatus*) (1, 11, 14, 24–34). Studies were included based on the availability of *cyp51A* genotype and the corresponding MICs obtained by *E*-test or broth microdilution performed according to CLSI and/or European Committee on Antimicrobial Susceptibility Testing (EUCAST) (Table S3).

### Next-generation sequencing

Genomic DNA was extracted using the EZ Tissue kit (Qiagen, Hilden, Germany) according to the manufacturer’s protocols after heat inactivation (95°C for 30 minutes) and a mechanical bead-beating step for fungal cell wall disruption. Sequencing libraries were prepared using the MagicPrep NGS System (Tecan, Männedorf, Switzerland) as previously described (35). Library quality was assessed using the High Sensitivity DNA Analysis kit (Agilent, Santa Clara, CA, USA), and quantification was determined using Qubit dsDNA High Sensitivity Assay kit (Thermo Fisher, Waltham, MA, USA). Paired-end sequencing (250 bp × 2) was performed on the Illumina MiSeq platform. NGS was performed on a total of 120 samples, including 11 subjected to repeat sequencing.

### Bioinformatic analysis

The bioinformatic analyses were performed using the Geneious Prime software (Geneious, Auckland, New Zealand). Paired sequencing files were downloaded from BaseSpace (Illumina) and imported into Geneious Prime version 11.

#### Assembly

The paired reads were mapped to a 1,941 bp assembly reference consisting of the *A. fumigatus* strain 237 14-alpha sterol demethylase (cyp51A) gene, complete CDS (National Center for Biotechnology Information AF338659), and 322 bp upstream of the CDS (promoter) (Fig. S1A). AF338659 is used as a wild-type reference by the CDC AR Bank and others (36, 37). The Geneious software performs mapped assembly according to the reference and continues with *de novo* assembly as permitted by continuous homology of the analyzed reads. The following quality metrics were recorded for the following using the contig nucleotide sequence: (i) total reads, (ii) assembled reads, (iii) mean coverage, and (iv) minimum coverage.

#### Tandem-repeat mutation analysis

Pairwise alignment was performed using the consensus nucleotide sequence and the complete AF338659 sequence. The “Find Motifs…” function was then used to identify the sequence “GAATCACGCGGTCCGGATGTGTGCTGAGCCGAAT” within the tandem-repeat mutations (TR34 or TR46). If present, the mean and minimum coverage of the mutations were recorded.

#### Non-synonymous mutation analysis

The CDS of reference AF338659 and the clinical isolate were translated to amino acid, and pairwise alignment was performed on the two protein sequences. For isolates with non-synonymous mutations, coverage (depth) of the mutant base(s) and the frequency of the mutation(s) were also recorded using statistics from the contig nucleotide sequence. The detailed bioinformatics protocol is provided in the supplemental document.

### Interpretation of results

Identified mutations and/or polymorphisms were interpreted according to interpretive criteria determined using the comprehensive data set above. Isolates whose polymorphisms were ambiguously associated with an AFST phenotype were considered indeterminate.

### Performance evaluation and analysis

Genotypic validation was performed in comparison to the results obtained by in-house Sanger sequencing at the UT Health at San Antonio FTL (19) or as published by the CDC AR Bank (20).

The percent agreement was calculated for the UCLA-sequenced sample set as the percentage of isolates whose *cyp51A* genotype was concordant with the expected AFST phenotype. Specifically, positive percent agreement (PPA) was calculated as the percentage of isolates with non-susceptible/non-wild-type phenotype and mutant *cyp51A* among the total number of isolates with non-wild-type *cyp51A*. Negative percent agreement (NPA) was calculated as the percentage of isolates with susceptible/wild-type phenotype and wild-type *cyp51A* among the total number of isolates with non-mutant *cyp51A*.

Geometric mean and 95% confidence interval (CI) for the comprehensive data set were calculated using Prism software version 10 (GraphPad, La Jolla, CA, USA) to formulate the interpretive criteria.

Performance characteristics, including PPA, NPA, error, and indeterminate rates, were calculated utilizing the clinical subset. True positive was defined as an isolate with non-susceptible/non-wild-type phenotype and a *cyp51A* mutation designated as a resistance determinant in the assay interpretive criteria. True negative was defined as an isolate with susceptible/wild-type phenotype and a wild-type *cyp51A* or a polymorphism not designated as a resistance determinant in the assay interpretive criteria. Very major error (VME) is calculated as the discordance of a susceptible/wild-type genotype result on a phenotypically resistant/non-wild-type isolate. Major error (ME) is calculated as the discordance of a resistant/non-wild-type genotype result on a phenotypically susceptible/wild-type isolate. Minor error (mE) is calculated as the discordance of any predictive result (susceptible/wild type or resistant/non-wild type) on a phenotypically intermediate isolate—not applicable to posaconazole and itraconazole—for which an intermediate phenotype is not defined. Isolates with an indeterminate result were not included in the calculation of the performance characteristics.

## RESULTS

**Fig 1:**
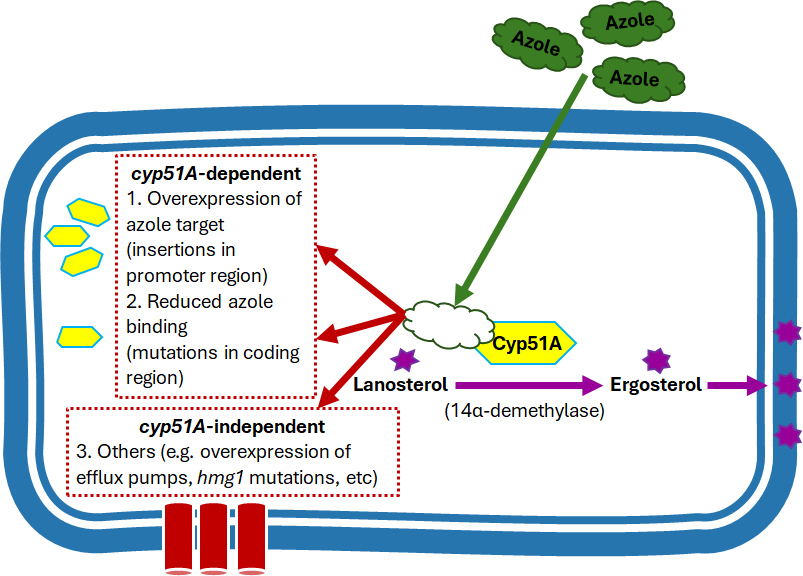
Triazole mechanism of action and resistance. Triazole drugs target the Cyp51A enzyme to inhibit the conversion of lanosterol to ergosterol. Known resistance mechanisms include *cyp51A*-dependent mutations such as (1) promoter mutations to result in overexpression of *cyp51A* and (2) CDS mutations to alter triazole affinity to the target, and *cyp51A*-independent mutations, including (3) upregulation of efflux pump genes, such as members of the major facilitator (MFS) and ATP-binding cassette (ABC) superfamilies, and mutations in the *hmg1* gene that affect a rate-limiting enzyme in the ergosterol biosynthetic pathway.

With the majority of *A. fumigatus* triazole resistance attributable to mutations in the *cyp51A* gene (Fig. 1) (14), we developed the triazole resistance assay to predict the AFST phenotype of clinically available triazole drugs. To initially validate the sequencing accuracy of the assay, we utilized a subset of 34 isolates from the CDC AR Bank with published *cyp51A* genotypes (*n* = 10) and the UT Health San Antonio FTL with independently analyzed *cyp51A* genes using an in-house Sanger sequencing protocol (*n* = 24) (Table 1) (19). The triazole resistance assay produced 97% (33 out of 34) complete genotypic concordance among the isolates, detecting 98% (53 out of 54) of all previously identified tandem-repeat mutations in the promoter region and non-synonymous mutations within the *cyp51A* CDS. The assay failed to detect a TR46 tandem-repeat mutation in one isolate due to insufficient assembly in the promoter region despite having ample high-quality sequences (~2 million reads) on the repeat run. This discrepancy, however, is not expected to result in a major error in resistance prediction, as TR46 alone was previously shown to have only a moderate effect on triazole susceptibility, and thus is likely to co-occur with other *cyp51A* mutations in resistant isolates (38).

**TABLE 1:** Genotypic-genotypic validation of the triazole resistance assay^*a*^

Sample no.	Sample ID	Detected *cyp51*A mutation(s)	Expected cyp*51A* mutation(s)	Complete concordance?	Mutation concordance?
1	AR-0731/ASP-01	TR34, L98H	TR34, L98H	Yes	2/2
2	AR-0732/ASP-02	TR34, L98H, S297T, F495I	TR34, L98H, S297T, F495I	Yes	4/4
3	AR-0733/ASP-03	TR34, L98H	TR34, L98H	Yes	2/2
4	AR-0734/ASP-04	TR34, L98H	TR34, L98H	Yes	2/2
5	AR-0735/ASP-05	TR34, L98H, S297T, F495I	TR34, L98H, S297T, F495I	Yes	4/4
6	AR-0736/ASP-06	None	None	Yes	0/0
7	AR-0737/ASP-07	None	None	Yes	0/0
8	AR-0738/ASP-08	None	None	Yes	0/0
9	AR-0739/ASP-09	None	None	Yes	0/0
10	AR-0740/ASP-10	None	None	Yes	0/0
11	UTHSA-DI20-081	TR34, L98H	TR34, L98H	Yes	2/2
12	UTHSA-DI20-082	G448S	G448S	Yes	1/1
13	UTHSA-DI20-084	None	None	Yes	0/0
14	UTHSA-DI20-086	G448S	G448S	Yes	1/1
15	UTHSA-DI20-091	None	None	Yes	0/0
16	UTHSA-DI20-096	None	None	Yes	0/0
17	UTHSA-DI20-098	G54R	G54R	Yes	0/0
18	UTHSA-DI20-100	TR34, L98H	TR34, L98H	Yes	2/2
19	UTHSA-DI20-102	TR46, Y121F, T289A	TR46, Y121F, T289A	Yes	3/3
20	UTHSA-DI20-103	Y121F, T289A, N512I	**TR46**, Y121F, T289A, N512I	**No**	**3/4**
21	UTHSA-DI20-106	TR46, Y121F, T289A	TR46, Y121F, T289A	Yes	3/3
22	UTHSA-DI20-108	None	None	Yes	0/0
23	UTHSA-DI20-112	TR34, L98H	TR34, L98H	Yes	2/2
24	UTHSA-DI20-113	F46Y, M172V, N248T, D255E, E427K	F46Y, M172V, N248T, D255E, E427K	Yes	5/5
25	UTHSA-DI20-115	F46Y, M172V, E427K	F46Y, M172V, E427K	Yes	3/3
26	UTHSA-DI20-128	TR34, L98H	TR34, L98H	Yes	2/2
27	UTHSA-DI20-132	M220K	M220K	Yes	1/1
28	UTHSA-DI20-134	TR46, Y121F, T289A	TR46, Y121F, T289A	Yes	3/3
29	UTHSA-DI20-180	Y121F	Y121F	Yes	1/1
30	UTHSA-DI21-184	F46Y, M172V, E427K	F46Y, M172V, E427K	Yes	3/3
31	UTHSA-DI21-187	M220I	M220I	Yes	1/1
32	UTHSA-DI21-188	M220R	M220R	Yes	1/1
33	UTHSA-DI20-192	P216L	P216L	Yes	1/1
34	UTHSA-DI21-193	P216L	P216L	Yes	1/1

^*a*^ Genotypic concordance was evaluated against a subset of isolates from the CDC AR Bank (*n* = 10) and UT Health at San Antonio FTL (*n* = 24) with known *cyp51A* mutations. Discordant result is marked in bold.

**Fig 2:**
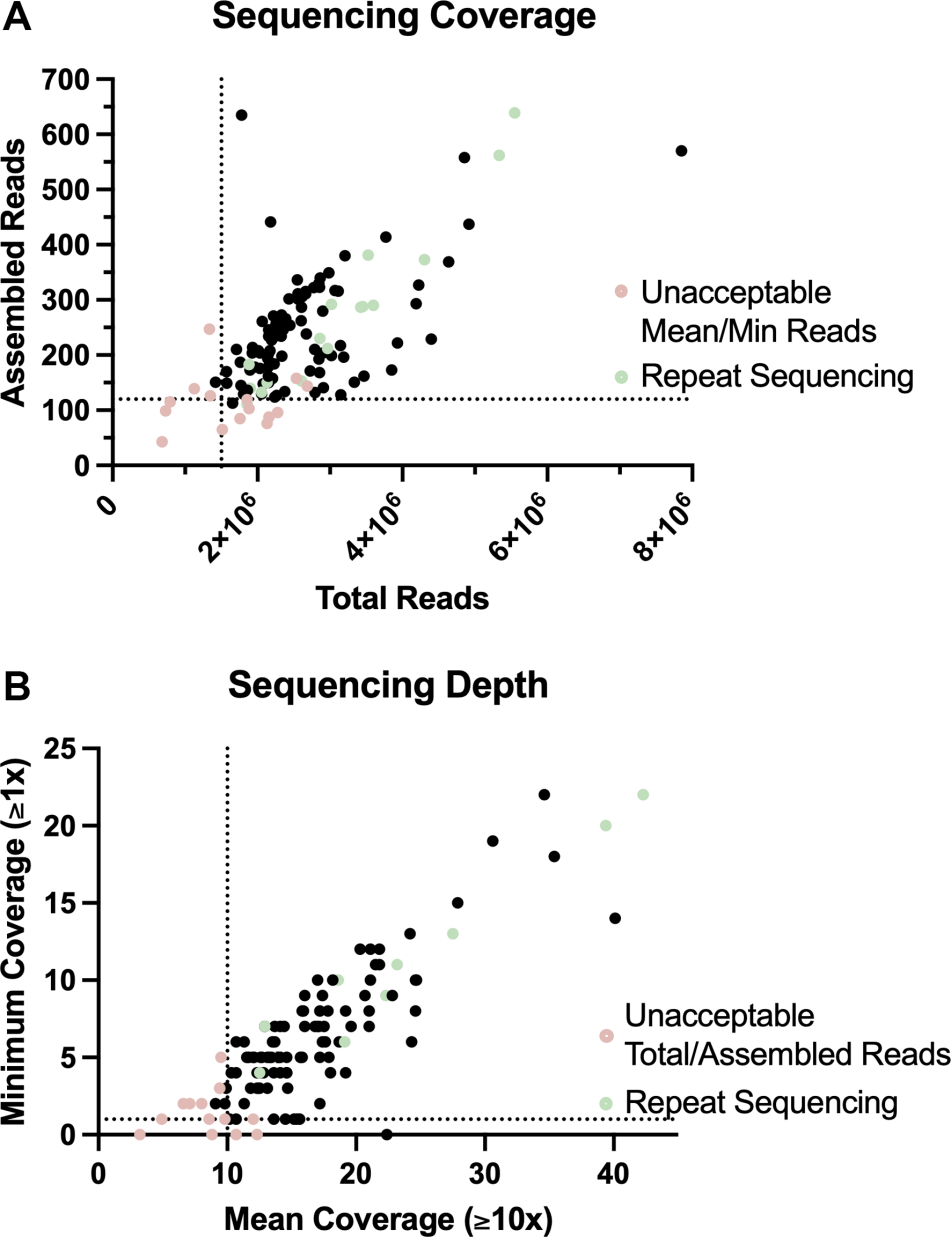
Sequencing quality metrics. (**A**) Correlation of total reads and assembled reads. Pink dots represent isolates with minimum and/or mean coverage that were below the designated thresholds of 1x and 10x, respectively. (**B**) Correlation of mean and minimum coverage (reads). Pink dots represent isolates with total and/or assembled reads that are below the designated thresholds of ≥1.5 × 10^6^x and ≥110x, respectively. (**A and B**) Green dots represent the corresponding repeat sequencing runs. Dotted lines represent the minimum threshold for each metric.

To establish sequencing quality metric thresholds for the assay, we sequenced an additional 75 clinical isolates from the UCLA Clinical Microbiology Lab (*n* = 30) and the UT Health San Antonio FTL (*n* = 45) (Table S1). Notably, using a previously published bioinformatic pipeline for WGS-based fungal identification, one isolate was ultimately identified as *Aspergillus lentulus*, a cryptic species within the *A. fumigatus* complex, and was excluded from further analyses (21). Assessment of sequencing reads demonstrated that a minimum of 110 assembled reads from a minimum of 1.5 × 10^6^ total reads correlated with complete coverage of the *cyp51A* gene (i.e., minimum coverage of ≥1× reads) with a mean coverage of ≥10× reads. Consistently, isolates with total and assembled reads that were initially below this cutoff resulted in passing coverage metrics in repeat sequencing with reads greater than the above thresholds (total reads ≥1.5 × 10^6^ and assembled reads ≥110) (Fig. 2).

**Fig 3:**
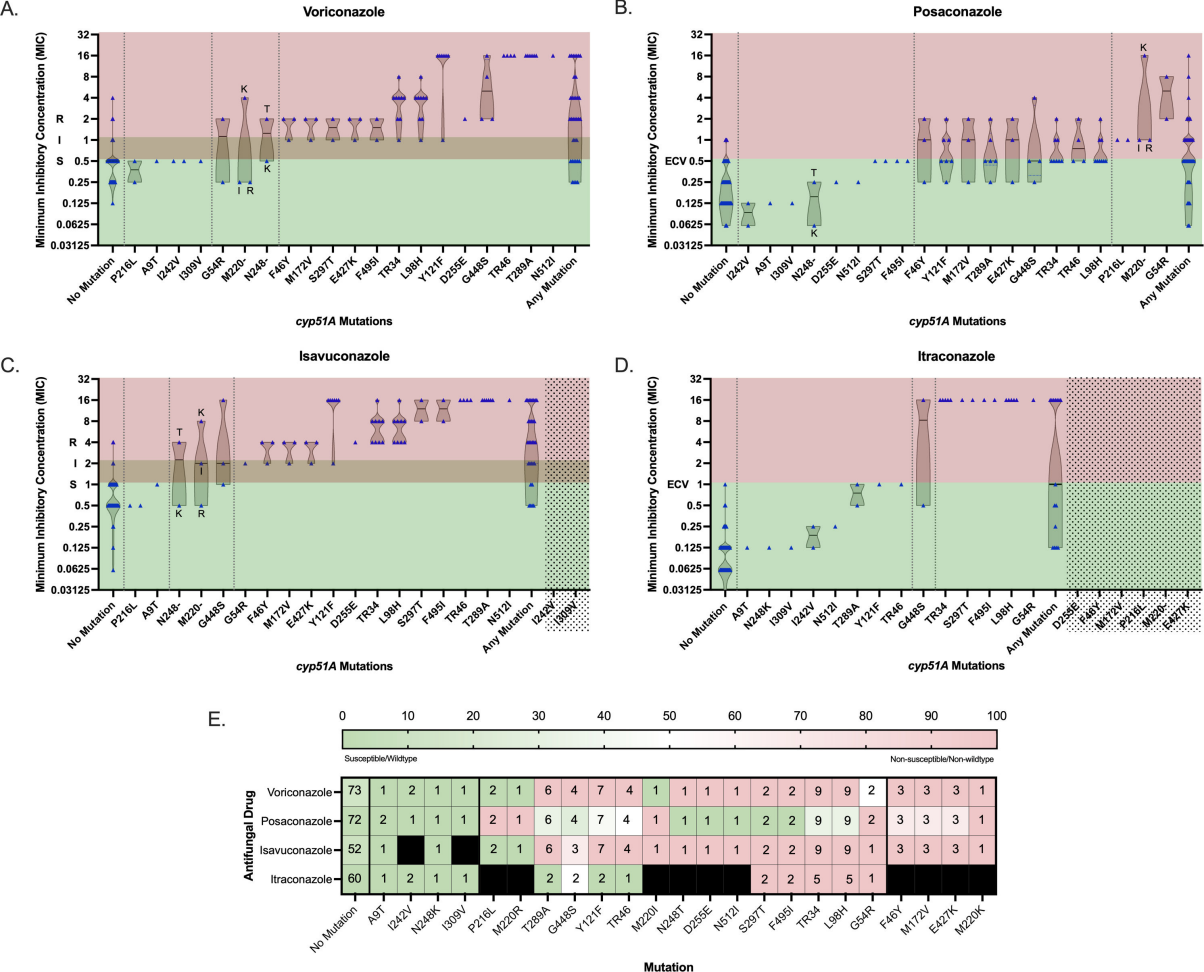
Phenotypic-genotypic comparison of the sequenced study set. (**A through D**) MICs are plotted against the individual mutation(s) detected in the isolate. For isolates with multiple detected mutations, the MICs are plotted accordingly in all the corresponding mutations. A truncated violin plot is overlaid with the individual data points. Dashes in mutations represent variable mutant amino acids. The specific corresponding mutant amino acids are noted adjacent to the data point. Dotted lines separate the *cyp51A* genotypes according to wild type (“no mutation”), universally susceptible/wild type, mixed, and universally resistant/non-wild type. Mutations with no corresponding MICs are marked with a gray box. The susceptible, intermediate, and resistant ranges are noted for voriconazole according to current CLSI guidelines. The ECVs are noted for posaconazole, isavuconazole, and itraconazole according to recently published ECVs. (**E**) The PPA of a non-susceptible AFST phenotype for each mutation is designated by the color heatmap and sorted according to overall PPA. Bolded lines separate the *cyp51A* genotypes according to wild type (no mutation), universally susceptible/wild type, mixed, and majority (>50%) resistant/non-wild type. The values listed in each cell correspond to the count of non-susceptible isolates in each category. Black cells correspond to mutant isolates whose MICs were not available.

Having established the working parameters for the triazole resistance assay, we next sought to evaluate the integrity of the genotypic results in predicting the outcome of the gold-standard phenotypic AFST for voriconazole, posaconazole, itraconazole, and isavuconazole. Currently, voriconazole and isavuconazole have interpretive criteria (breakpoints) for *A. fumigatus* from CLSI (22, 23). Posaconazole and itraconazole were phenotypically discriminated as wild type or non-wild type using published ECVs (Fig. 3A through D) (24).

First, we evaluated the raw NPA and PPA between a *cyp51A* genotype and the expected phenotype for each drug (Table 2), where any mutation and/or polymorphism was expected to produce a non-susceptible/non-wild-type MIC. The NPA for each triazole drug ranged from 88% to 100%. The highest NPAs were for posaconazole and itraconazole, in which nearly all the isolates with wild-type *cyp51A* (99%–100%) had MICs less than or equal to their respective ECVs (Fig. 3B and D). In contrast, the PPA of a mutant gene with a non-wild-type MIC was much lower and more variably correlated with the predicted AFST phenotype among the different azoles. While reasonably 71% and 80% of mutant *cyp51A* isolates had non-susceptible voriconazole and isavuconazole MICs, respectively, much lower proportions were observed for posaconazole (46%) and itraconazole (47%). It is worth noting that limited phenotypic-genotypic data were available for itraconazole.

**TABLE 2:** Raw percent agreement of UCLA-sequenced sample set^*a*^

Performance	NPA	PPA
Voriconazole	88% (64)	71% (25)
Posaconazole	99% (71)	46% (16)
Isavuconazole	92% (48)	80% (24)
Itraconazole	100% (60)	47% (7)

^*a*^ Raw negative percent agreement (NPA) and positive percent agreement (PPA), respectively, are calculated for the UCLA-sequenced sample set where any mutation and/or polymorphism is expected to produce a non-susceptible/non-wild-type MIC. The values in parentheses represent the count of isolates in each category.

To further discern the individual associations between a specific mutation and the corresponding triazole MIC, we also assessed the PPA of each mutation for each triazole drug (Fig. 3E). In more than half of the detected mutations, the genotypic-phenotypic agreements were highly variable among the triazole drugs, with wild-type posaconazole AFST associating frequently with *cyp51A* mutations. This observation highlights the heterogenous relationships between mutations in the *cyp51A* gene and a non-wild-type phenotype.

On the other hand, there were several noted mutations (A9T, I242V, I309V, and N248K), albeit with lower frequencies of detection (Table S2), in which 100% (five out of five) of the isolates demonstrated consistently susceptible voriconazole and isavuconazole phenotypes and MICs below their respective ECVs for posaconazole and itraconazole. This finding indicates that these mutations may be naturally occurring genetic polymorphisms and not independently causative of drug resistance. Likewise, well-documented mutations, such as F46Y, M172V, E4227K, and M220K, strongly correlated with elevated MICs in >50% of the corresponding mutant isolates across all four clinical triazole drugs (39). Lastly, consistent with prior reports, voriconazole and isavuconazole appear to be similarly impacted by the *cyp51A* mutations due to their structural similarity (19, 40, 41).

**Fig 4:**
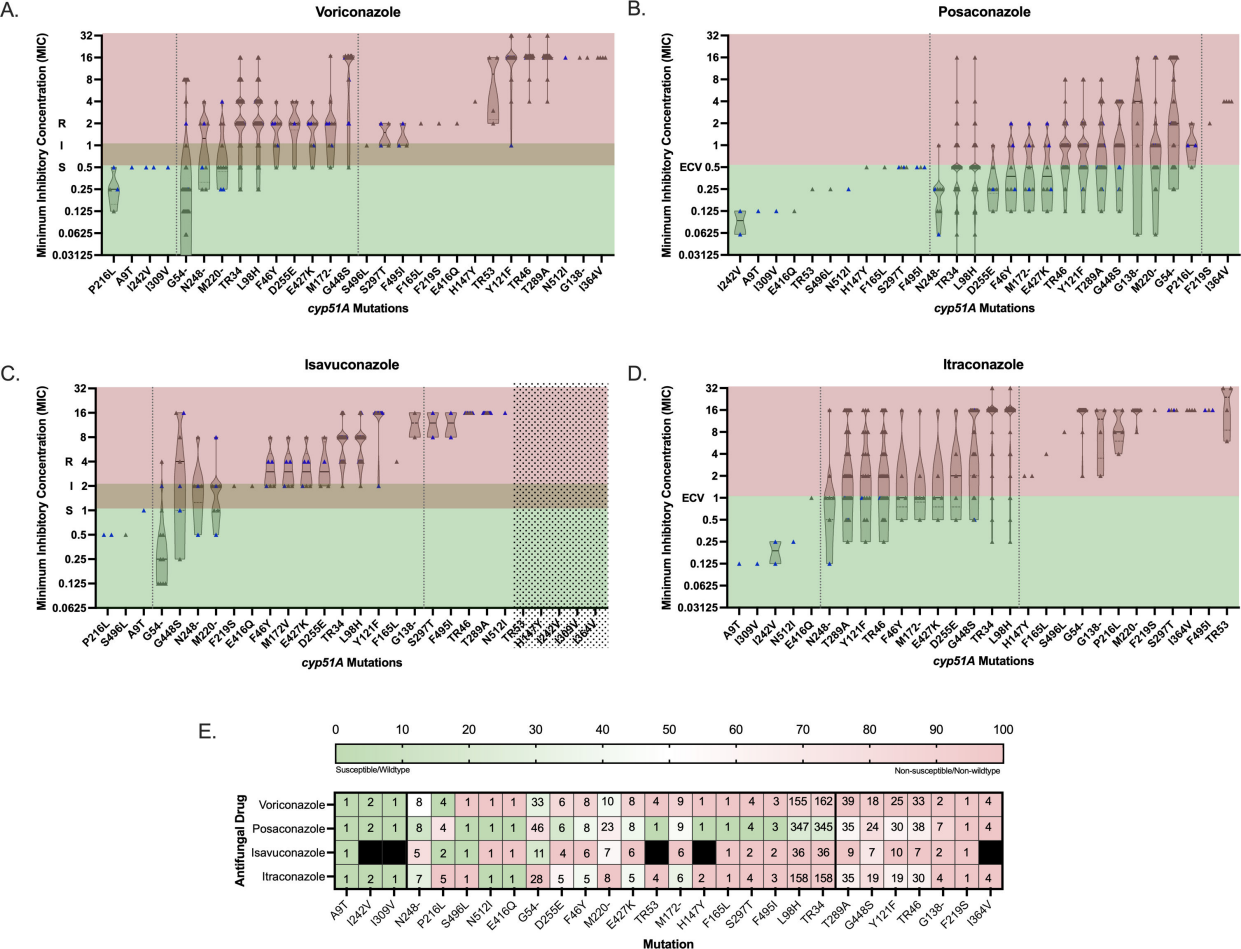
Phenotypic-genotypic comparison of the comprehensive data set. (**A through D**) MICs are plotted against the individual mutation(s) detected in the isolate. For isolates with multiple detected mutations, the MICs are plotted accordingly in all the corresponding mutations. The UCLA-sequenced data set is marked in blue. A truncated violin plot is overlaid on the individual data points. Dotted lines separate the *cyp51A* genotypes according to universally susceptible/wild type, mixed, and universally resistant/non-wild type. The susceptible, intermediate, and resistant ranges are noted for voriconazole according to current CLSI guidelines. The ECVs are noted for posaconazole, isavuconazole, and itraconazole according to recently published ECVs. (**E**) The PPA of a non-susceptible AFST phenotype for each mutation is designated by the color heatmap and sorted according to the overall PPA. Bold lines separate the *cyp51A* genotypes according to universally susceptible/wild type, mixed, and majority (50%) resistant/non-wild type. The values listed in each cell correspond to the count of non-susceptible isolate(s) in each category. Black cells correspond to mutant isolates whose MICs were not available. (**A through E**) Dashes in mutations represent variable mutant amino acids.

Given the significant variability in the resistance associations of *cyp51A* mutations among our study set and the indication of common naturally occurring polymorphisms, we sought to fortify our database and create a comprehensive data set by supplementing the UCLA-sequenced isolates with previously published peer-reviewed studies (*n* = 14 studies, Table S3) (1, 11, 14, 24–34). Similar to the results of the UCLA-sequenced sample set alone, the comprehensive data set corroborated the variable associations between a given *cyp51A* mutation and triazole resistance (Fig. 4). Of note, several mutations are associated with elevated MICs across all four clinical triazole drugs in more than 50% of documented mutants, designating them as potential predictors of pan-azole resistance (T289A, G448S, Y121F, TR46, G138−, F219S, and I364V) (Fig. 4E). In contrast, A9T, I242V, or I309V are frequently correlated with wild-type MICs, indicating possible wild-type genetic polymorphisms (Fig. 4E).

**Fig 5:**
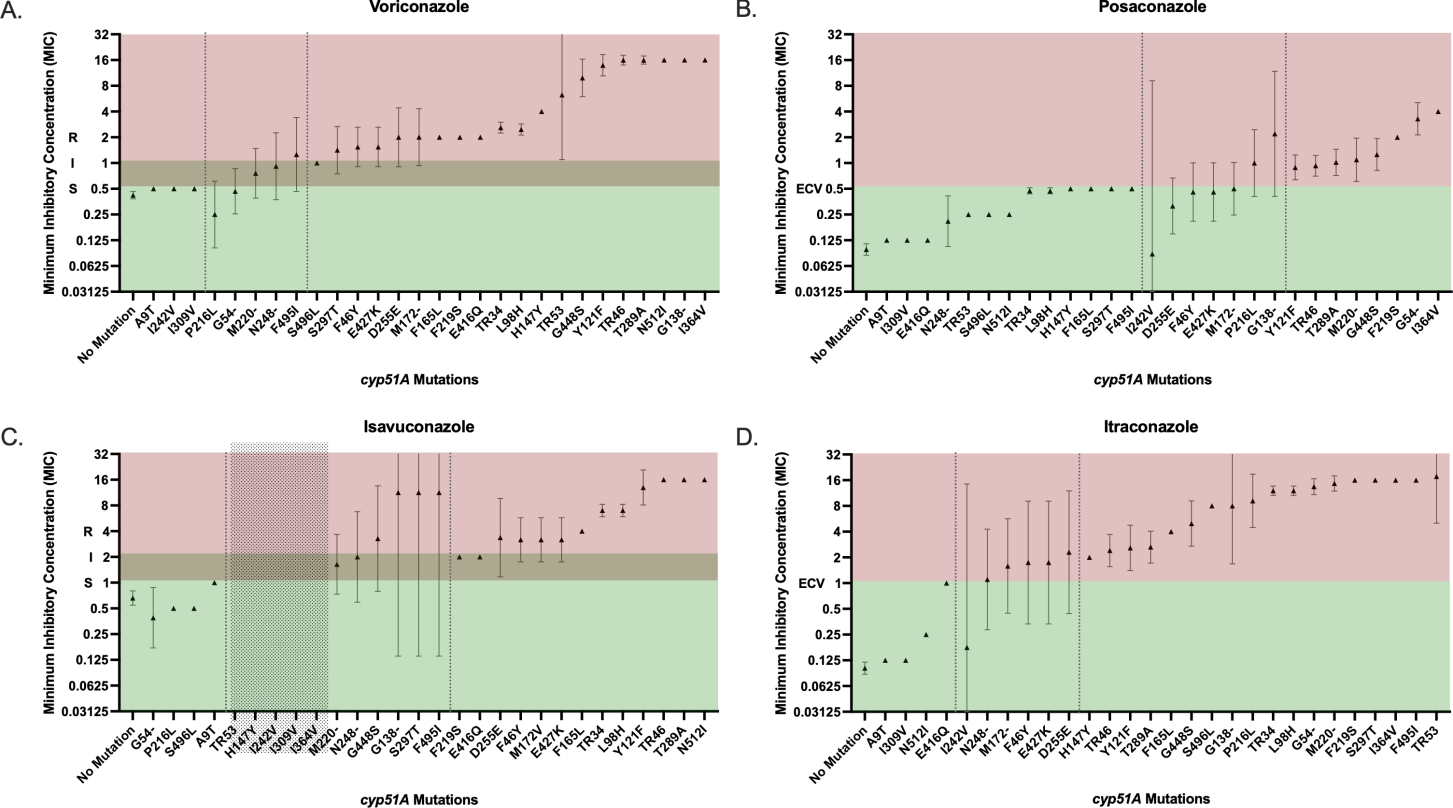
Analysis of comprehensive data set. (**A through D**) Mean and 95% CI are plotted for the comprehensive data set. For isolates with multiple detected mutations, the MICs are plotted accordingly in all the corresponding mutations. Dotted lines separate the *cyp51A* genotypes according to universally susceptible/wild type, mixed, and universally resistant/non-wild type. The susceptible, intermediate, and resistant ranges are noted for voriconazole according to current CLSI guidelines. The ECVs are noted for posaconazole, isavuconazole, and itraconazole according to recently published ECVs. Dashes in mutations represent variable mutant amino acids.

Calculation of the geometric mean and 95% CI of each mutation/polymorphism for each drug ultimately provided superior discrimination between the mutations that are predicted to confer likely resistant/non-wild-type MICs, and the *cyp51A* genotypes that may yield elevated MICs yet are not independently sufficient to predict categorical drug resistance (Fig. 5A through D). Interestingly, a subset of mutations was indeterminate predictors of triazole phenotype, in which the mutant isolates variably produced MICs that widely spanned the susceptible/wild-type and non-susceptible/non-wild-type categories (Fig. 5A through D).

Collectively, using the comprehensive data set comprised of the UCLA-sequenced sample set and previously published isolates, we established interpretive criteria for the triazole resistance assay (Table 3). First, for voriconazole, isolates with a given *cyp51A* mutation were predicted to be “resistant” if the corresponding geometric mean MICs and 95% CIs were fully within the non-susceptible (intermediate or resistant) categories as defined by the current CLSI guidelines (22, 42). In converse, *cyp51A*-wild-type isolates or those with *cyp51A* polymorphism(s) whose corresponding geometric MICs and 95% CIs were fully within the susceptible range were predicted to be “susceptible” to voriconazole. Of note, the geometric mean and 95% CI for wild-type isolates (“no mutation”) was calculated using only the clinical subset to avoid bias toward phenotypically non-susceptible isolates with wild-type *cyp51A* that are frequently included in published studies. For isolates with a given mutation in which the geometric mean MIC and 95% CI ranged between susceptible and non-susceptible, the results of the triazole resistance assay were deemed indeterminate, with the assay unable to predict the likely phenotype.

**TABLE 3:** Interpretive criteria of triazole resistance assay^*a*^

Domain/position	Mutation/polymorphism	Result interpretation			
		Voriconazole	Posaconazole	Isavuconazole	Itraconazole
None		Susceptible	Wild type	Susceptible	Wild type
Promoter	TR34	Resistant	Wild type	Resistant	Non-wild type
Promoter	TR46	Resistant	Non-wild type	Resistant	Non-wild type
Promoter	TR53	Resistant	Wild type	Indeterminate	Non-wild type
9	A9T	Susceptible	Wild type	Susceptible	Wild type
46	F46Y	Resistant	Indeterminate	Resistant	Indeterminate
54	G54−	Indeterminate	Non-wild type	Susceptible	Non-wild type
98	L98H	Resistant	Wild type	Resistant	Non-wild type
121	Y121F	Resistant	Non-wildtype	Resistant	Non-wild type
138	G138−	Resistant	Indeterminate	Indeterminate	Non-wild type
147	H147Y	Resistant	Wild type	Indeterminate	Non-wild type
165	F165L	Resistant	Wild type	Resistant	Non-wild type
172	M172−	Resistant	Indeterminate	Resistant	Indeterminate
216	P216L	Indeterminate	Indeterminate	Susceptible	Non-wild type
219	F219S	Resistant	Non-wild type	Resistant	Non-wild type
220	M220−	Indeterminate	Non-wild type	Indeterminate	Non-wild type
242	I242V	Susceptible	Indeterminate	Indeterminate	Indeterminate
248	N248-	Indeterminate	Wild type	Indeterminate	Indeterminate
255	D255E	Resistant	Indeterminate	Resistant	Indeterminate
297	S297T	Resistant	Wild type	Indeterminate	Non-wild type
289	T289A	Resistant	Non-wild type	Resistant	Non-wild type
309	I309V	Susceptible	Wild type	Indeterminate	Wild type
364	I364V	Resistant	Non-wild type	Indeterminate	Non-wild type
416	E416Q	Resistant	Wild type	Resistant	Wild type
427	E427K	Resistant	Indeterminate	Resistant	Indeterminate
448	G448S	Resistant	Non-wild type	Indeterminate	Non-wild type
495	F495I	Indeterminate	Wild type	Indeterminate	Non-wild type
496	S496L	Resistant	Wild type	Susceptible	Non-wild type
512	N512I	Resistant	Wild type	Resistant	Wild type

^*a*^ Interpretive criteria based on geometric mean and 95% CI of the comprehensive data set. Dashes in mutations represent variable mutant amino acids.

Likewise, the interpretive criteria for the other triazole drugs were similarly determined using the same guidelines, with posaconazole and itraconazole being discriminated as non-wild type, wild type, or indeterminate, respectively. For isolates with multiple detected mutations/polymorphisms, the overall predictive result was conservatively reported according to the most resistance-conferring mutation.

Lastly, to determine the most appropriately representative performance characteristics of the triazole resistance assay, we utilized the clinical subset (*n* = 75) among the UCLA-sequenced samples, which is a set of unbiased and blinded isolates randomly collected upon clinical requests by a physician for AFST. The samples were sequenced and analyzed according to the established criteria above (Table 3), and the adjusted performance characteristics were calculated based on concordance between the predicted and actual AFST result (Table 4). The triazole resistance assay produced a remarkable NPA (also calculated as specificity) of ≥95%, while the PPA (also calculated as sensitivity) varied widely among the triazole drugs (Table 5). Notably, none of the discordant results were categorically VMEs and were mostly among MEs and mEs. Additionally, the rates of indeterminate results were minimal at 3%–5%.

**TABLE 4:** Clinical subset used for determination of performance characteristics of the triazole resistance assay^*a*^

Sample no.	Accession no.	*cyp51A* genotype	Voriconazole		Posaconazole		Isavuconazole		Itraconazole	
			MIC (µg/mL)	Triazole resistance assay result	MIC (µg/mL)	Triazole resistance assay result	MIC (µg/mL)	Triazole resistance assay result	MIC (µg/mL)	Triazole resistance assay result
1	UCLA-001	Wild type	0.5	Susceptible	0.06	Wild type	0.5	Susceptible	N/A	N/A
2	UCLA-002	Wild type	0.25	Susceptible	0.03	Wild type	0.5	Susceptible	N/A	N/A
3	UCLA-003	Wild type	0.5	Susceptible	0.06	Wild type	1	Susceptible	N/A	N/A
4	UCLA-004	Wild type	*1*	Susceptible*	0.25	Wild type	*2*	Susceptible*	N/A	N/A
5	UCLA-005	G448S	**16**	**Resistant**	**4**	**Non-wild type**	(16)	Indeterminate	**16**	**Non-wild type**
6	UCLA-006	Wild type	0.25	Susceptible	0.06	Wild type	0.5	Susceptible	0.25	Wild type
7	UCLA-007	Wild type	0.25	Susceptible	0.06	Wild type	0.5	Susceptible	N/A	N/A
8	UCLA-008	Wild type	0.5	Susceptible	0.06	Wild type	0.5	Susceptible	0.25	Wild type
9	UCLA-009	Wild type	0.5	Susceptible	0.125	Wild type	0.5	Susceptible	0.5	Wild type
10	UCLA-010	Wild type	0.5	Susceptible	0.125	Wild type	0.5	Susceptible	1	Wild type
11	UCLA-011	Wild type	0.5	Susceptible	0.125	Wild type	0.5	Susceptible	1	Wild type
12	UCLA-012	Wild type	0.25	Susceptible	0.06	Wild type	0.5	Susceptible	0.125	Wild type
13	UCLA-013	Wild type	0.25	Susceptible	0.125	Wild type	0.5	Susceptible	0.5	Wild type
14	UCLA-014	Wild type	0.5	Susceptible	0.5	Wild type	0.5	Susceptible	1	Wild type
15	UCLA-015	Wild type	0.25	Susceptible	0.03	Wild type	0.5	Susceptible	0.25	Wild type
16	UCLA-016	Wild type	0.25	Susceptible	0.125	Wild type	0.5	Susceptible	0.125	Wild type
17	UCLA-017	Wild type	0.25	Susceptible	0.03	Wild type	0.06	Susceptible	0.06	Wild type
18	UCLA-018	Wild type	0.5	Susceptible	0.06	Wild type	0.5	Susceptible	0.5	Wild type
19	UCLA-019	Y121F, T289A	**16**	**Resistant**	0.5	Non-wild type*	**16**	**Resistant**	0.5	Non-wild type*
20	UCLA-020	Wild type	0.5	Susceptible	0.06	Wild type	1	Susceptible	N/A	N/A
21	UCLA-021	Wild type	0.5	Susceptible	0.25	Wild type	1	Susceptible	N/A	N/A
22	UCLA-022	Wild type	0.5	Susceptible	0.25	Wild type	0.5	Susceptible	1	Wild type
23	UCLA-023	Wild type	0.5	Susceptible	0.125	Wild type	1	Susceptible	N/A	N/A
24	UCLA-024	TR46, Y121F, T289A	**16**	**Resistant**	0.5	Non-wild type*	**16**	**Resistant**	1	Non-wild type*
25	UCLA-025	Wild type	0.5	Susceptible	0.125	Wild type	1	Susceptible	0.25	Wild type
26	UCLA-026	Wild type	*1*	Susceptible*	0.125	Wild type	0.5	Susceptible	0.25	Wild type
27	UCLA-027	Wild type	*1*	Susceptible*	0.06	Wild type	1	Susceptible	N/A	N/A
28	UCLA-028	Wild type	0.5	Susceptible	0.06	Wild type	1	Susceptible	0.5	Wild type
29	UCLA-029	Wild type	0.5	Susceptible	0.125	Wild type	0.5	Susceptible	0.125	Wild type
30	UCLA-030	Wild type	0.5	Susceptible	0.06	Wild type	1	Susceptible	0.5	Wild type
31	UTHSA-DI24-307	Wild type	0.5	Susceptible	0.125	Wild type	1	Susceptible	0.125	Wild type
32	UTHSA-DI24-308	Wild type	0.5	Susceptible	0.125	Wild type	1	Susceptible	0.5	Wild type
33	UTHSA-DI24-309	Wild type	0.5	Susceptible	N/A	N/A	N/A	N/A	0.125	Wild type
34	UTHSA-DI24-310	Wild type	0.25	Susceptible	0.06	Wild type	0.5	Susceptible	0.5	Wild type
35	UTHSA-DI24-311	Wild type	0.5	Susceptible	0.125	Wild type	N/A	N/A	0.25	Wild type
36	UTHSA-DI24-312	Wild type	0.5	Susceptible	0.125	Wild type	1	Susceptible	0.125	Wild type
37	UTHSA-DI24-313	Wild type	0.5	Susceptible	0.125	Wild type	1	Susceptible	0.125	Wild type
38	UTHSA-DI24-314	Wild type	0.5	Susceptible	0.06	Wild type	N/A	N/A	0.125	Wild type
39	UTHSA-DI24-315	Wild type	0.25	Susceptible	0.06	Wild type	0.5	Susceptible	0.125	Wild type
40	UTHSA-DI24-316	Wild type	0.5	Susceptible	0.06	Wild type	N/A	N/A	0.125	Wild type
41	UTHSA-DI24-317	I242V	0.5	Susceptible	(0.06)	Indeterminate	N/A	N/A	(0.125)	Indeterminate
42	UTHSA-DI24-318	G54R	(0.25)	Indeterminate	**2**	**Non-wild type**	N/A	N/A	**16**	**Non-wild type**
43	UTHSA-DI24-319	Wild type	0.25	Susceptible	0.125	Wild type	0.25	Susceptible	0.5	Wild type
44	UTHSA-DI24-320	Wild type	0.25	Susceptible	0.125	Wild type	N/A	N/A	0.5	Wild type
45	UTHSA-DI24-321	G448S	**8**	**Resistant**	0.5	Non-wild type*	N/A	N/A	0.5	Non-wild type*
46	UTHSA-DI24-322	Wild type	0.5	Susceptible	0.125	Wild type	1	Susceptible	0.125	Wild type
47	UTHSA-DI24-323	I309V	0.5	Susceptible	0.125	Wild type	N/A	N/A	0.125	Wild type
48	UTHSA-DI24-325	A9T	0.5	Susceptible	0.125	Wild type	1	Susceptible	0.125	Wild type
49	UTHSA-DI24-326	Wild type	0.25	Susceptible	0.06	Wild type	0.5	Susceptible	0.25	Wild type
50	UTHSA-DI24-327	Wild type	0.5	Susceptible	0.25	Wild type	N/A	N/A	0.25	Wild type
51	UTHSA-DI24-328	Wild type	0.25	Susceptible	0.125	Wild type	N/A	N/A	0.5	Wild type
52	UTHSA-DI24-329	Wild type	0.5	Susceptible	0.06	Wild type	N/A	N/A	0.25	Wild type
53	UTHSA-DI24-330	Wild type	0.5	Susceptible	0.125	Wild type	1	Susceptible	0.125	Wildtype
54	UTHSA-DI24-331	Wild type	0.5	Susceptible	0.125	Wild type	N/A	N/A	0.125	Wild type
55	UTHSA-DI24-332	Wild type	0.5	Susceptible	0.25	Wild type	1	Susceptible	0.25	Wild type
56	UTHSA-DI24-333	I242V	0.5	Susceptible	(0.125)	Indeterminate	N/A	N/A	(0.25)	Indeterminate
57	UTHSA-DI24-334	Wild type	0.25	Susceptible	0.06	Wild type	N/A	N/A	0.25	Wild type
58	UTHSA-DI24-335	Wild type	0.5	Susceptible	0.06	Wild type	N/A	N/A	0.125	Wild type
59	UTHSA-DI24-336	Wild type	*1*	Susceptible*	0.125	Wild type	N/A	N/A	0.25	Wild type
60	UTHSA-DI24-337	Wild type	0.5	Susceptible	0.125	Wild type	N/A	N/A	0.25	Wild type
61	UTHSA-DI24-338	Wild type	0.5	Susceptible	0.25	Wild type	1	Susceptible	0.5	Wild type
62	UTHSA-DI24-339	Wild type	0.5	Susceptible	0.125	Wild type	1	Susceptible	0.25	Wild type
63	UTHSA-DI24-340	Wild type	0.5	Susceptible	0.125	Wild type	0.5	Susceptible	0.25	Wild type
64	UTHSA-DI24-341	Wild type	0.5	Susceptible	0.06	Wild type	0.5	Susceptible	0.125	Wild type
65	UTHSA-DI24-342	N248K	(0.5)	Indeterminate	0.06	Wild type	(0.5)	Indeterminate	(0.125)	Indeterminate
66	UTHSA-DI24-343	Wild type	0.25	Susceptible	0.06	Wild type	N/A	N/A	0.125	Wild type
67	UTHSA-DI24-344	Wild type	0.25	Susceptible	0.06	Wild type	N/A	N/A	0.125	Wild type
68	UTHSA-DI24-345	Wild type	0.25	Susceptible	0.06	Wild type	N/A	N/A	0.125	Wild type
69	UTHSA-DI24-346	Wild type	0.5	Susceptible	0.06	Wild type	N/A	N/A	0.5	Wild type
70	UTHSA-DI24-347	Wild type	0.5	Susceptible	0.06	Wild type	N/A	N/A	0.125	Wild type
71	UTHSA-DI24-348	Wild type	0.25	Susceptible	0.06	Wild type	0.5	Susceptible	0.25	Wild type
72	UTHSA-DI24-349	Wild type	0.5	Susceptible	0.125	Wild type	N/A	N/A	0.125	Wild type
73	UTHSA-DI24-350	Wild type	0.5	Susceptible	0.25	Wild type	N/A	N/A	0.125	Wild type
74	UTHSA-DI24-351	Wild type	0.25	Susceptible	0.5	Wild type	N/A	N/A	0.06	Wild type
75	UTHSA-DI24-324	*A. lentulus*								

^*a*^ Resistant/non-wild-type MIC and “resistant” triazole resistance assay results are shown in bold. “Intermediate” voriconazole and isavuconazole MIC are shown in italics. Corresponding MICs of isolates with indeterminate triazole resistance assay results were excluded from performance analyses and are noted in parentheses. N/A represents not available (MIC) or not applicable (triazole resistance assay result). Discordant MICs and triazole resistance assay results are noted with an asterisk (*).

**TABLE 5:** Performance characteristics of the triazole resistance assay^*a*^

Performance	True		False		Error			NPA (%)	PPA (%)	Indeterminate (rate, %)
	Positive	Negative	Positive	Negative	VME	ME	mE			
Voriconazole	4	64	0	4	0	0	4	100	50	2 (3)
Posaconazole	2	66	3	0	0	3	N/A	96	100	2 (3)
Isavuconazole	2	43	0	1	0	0	1	100	67	2 (4)
Itraconazole	2	57	3	0	0	3	N/A	95	100	3 (5)

^*a*^ Adjusted performance characteristics (PPA, NPA, PPV, and NPV) and errors are calculated according to the concordance between the AFST phenotype and the predicted triazole phenotype according to the *cyp51A* genotype interpretive criteria. N/A in minor error (mE) represents not applicable due to unavailability of intermediate phenotype categorization.

## DISCUSSION

The alarming rise in triazole resistance among clinical *A. fumigatus* isolates is challenging the current paradigm of antifungal treatment and management. Accordingly, the need for AFST is becoming increasingly more common, particularly in critical cases of aspergillosis. Unfortunately, phenotypic AFST is limited by the inherent difficulty of its performance and interpretation, despite highly standardized guidelines. To accommodate the need for more rapid and clinically actionable results, we developed and validated the NGS-based triazole resistance assay analyzing the *cyp51A* gene as a predictive marker for triazole phenotype in *A. fumigatus* isolates.

The triazole resistance assay utilizes a WGS approach that offers several advantages. First, confirmation of species-level identification can be performed using a previously published analytical pipeline to appropriately identify *A. fumigatus sensu stricto* among other members of the *A. fumigatus* complex (21). The *cyp51A* gene for other members of *A. fumigatus sensu lato* is sufficiently non-homologous to be incompatible with the sequencing analysis of the assay and prompts further investigation (Fig. S1A, bottom panel) (40, 43). This feature may prove to be valuable in identifying cryptic species that may phenotypically resemble *A. fumigatus sensu stricto* but may warrant different clinical interpretation and/or patient management. Additionally, WGS will allow for future investigations of other triazole resistance-associated genes, for which at present time has limited publicly available data.

More notably, in laboratories already equipped with NGS platforms, in-house performance of the triazole resistance assay can offer a more expedited turnaround time compared to conventional AFST performed at a specialty reference laboratory. Currently, time to reporting at UCLA from the initial request of phenotypic AFST results ranges from 3 to 4 weeks, owing to both technical and procedural workflow hurdles with send-out testing. In contrast, in-house sequencing at the UCLA Molecular Microbiology & Pathogen Genomics (MMPG) laboratory is performed at least once a week, with a potential to accommodate a second run as necessary, culminating in a turnaround time of <10 days on average. Indeed, optimization of the sequencing workflow that incorporates commercially available tools has been previously shown to not only decrease hands-on time without compromising quality but also support the feasibility of in-house sequencing even at smaller scales (35).

Correspondingly, the sequencing requirements (i.e., depth and coverage) of the triazole resistance assay are comparable to previously published sequencing assays similarly utilizing gene markers for predicting drug resistance. Thus, this minimizes the need for significant optimization in the performing laboratory (44–46). Based on our validation, we determined that a minimum of 1.5 million reads (2 × 250 bp) and a minimum mean coverage of 10× reads were sufficient for this genotyping assay, thus allowing for the triazole resistance assay to be seamlessly incorporated in existing culture-dependent WGS workflows sharing similar sequence quality control criteria. Lastly, the use of commercially available software with a user-friendly graphical user interface for the post-analytical phase further allows for ease of adaptation in laboratories with existing sequencing assays.

Nevertheless, there are some limitations worth discussing for the triazole resistance assay. Perhaps the most apparent is regarding some of the suboptimal performance characteristics of the assay. First, the PPAs of the assay for voriconazole and isavuconazole resistance prediction are 50% and 67%, respectively. Part of this limitation may be attributed to the limited counts of phenotypically resistant isolates included in the UCLA clinical subset, particularly for isavuconazole. However, a pertinent finding is that for the discordant isolates predicted to be susceptible to voriconazole (four out of four) and isavuconazole (one out of one) according to its wild-type *cyp51A* gene, the phenotypic MICs were only moderately elevated at 1 and 2 µg/mL, respectively, which are interpreted as “intermediate” according to current CLSI breakpoints. The intermediate category is intended to provide a buffer zone for phenotypic AFST that is “necessary to avoid major and very major errors that may occur, given the inherent variability in the *in vitro* testing method” (42). Further, “available data do not permit isolates with [MIC] results in the intermediate range to be clearly categorized as either ‘susceptible’ or ‘resistant’” (42). Thus, isolates with categorically intermediate susceptibility to voriconazole and isavuconazole are not precluded from the possibility of clinical efficacy within the host that may contradict the *in vitro* phenotypic MIC in support of the genotypic results. Accordingly, these errors are only deemed to be mEs. Alternative result categorization as “Resistant” and “Non-resistant” may overcome such ambiguous non-discordance to yield PPAs of 100% across all 4 triazole drugs. Further clinical evaluation of these isolates is necessary to reconcile the clinical significance of such discrepancies.

Consistently, given the infrequency of resistant/non-wild-type isolates (3%–8%) due to its limited availability for the study, a robust representation of the performance characteristics of the triazole resistance assay is difficult to ascertain. However, in a presumption of the phenotypic resistance/non-wild-type prevalence of up to 26% noted above, high positive predictive values of 100/89/100/88 and negative predictive values of 85/100/90/100 (for voriconazole, posaconazole, isavuconazole, and itraconazole, respectively) can be anticipated (14). While the experimental design of our assay validation is not sufficiently powered to assess mutational prevalence, our data support further investigations into mutation-based antifungal resistance in *A. fumigatus* to accurately appreciate the clinical performance of the assay.

Despite its limitations in performance, a notable feature of the triazole resistance assay is its remarkable specificity (NPA) of at least 95% for any given drug. Clinically, a common challenge among patients who fail to respond to triazole therapy is discriminating between true microbiological resistance of the isolate and poor outcome due to the patient’s declining capacity to fight an infection (i.e., immunosuppression). In such context, accurately identifying susceptible/wild-type isolates—and thus, pointing toward the latter as the likely contributing factor to response failure—can be paramount in the clinical decision-making on whether to change the therapeutic regimen. Accordingly, despite the suboptimal performance in identifying isolates with likely phenotypic resistance (particularly for voriconazole and isavuconazole), it may be clinically sufficient for an assay to identify isolates with likely susceptible MICs. Further studies incorporating clinical data and patient outcomes are needed to effectively establish the optimal clinical utility and performance of the assay in a given population.

Overall, our triazole resistance assay capitalized upon more than 1,800 collected data points to produce rapid and clinically actionable results. As demonstrated by the highly variable PPA of a mutant *cyp51A* genotype and a resistant phenotype, it was critical and necessary to utilize a large and comprehensive data set to most accurately approximate the natural impact of mutations on drug susceptibility, particularly for those that have not been well characterized in literature. Thus, while limitations exist with some of the performance characteristics of the triazole resistance assay, further expansion of the database will undoubtedly improve its performance and optimize the predictive value of the results.

## Data Availability

Genomic data are available upon request.

## SUPPLEMENTAL MATERIAL

Supplemental material jcm.00291-25-s0001.docx Fig. S1, Tables S1 to S3, and bioinformatics protocol.

ASM does not own the copyrights to Supplemental Material that may be linked to, or accessed through, an article. The authors have granted ASM a non-exclusive, world-wide license to publish the Supplemental Material files. Please contact the corresponding author directly for reuse.
