# Supplementary material for: Development and validation of next-generation sequencing-based clinical test for triazole resistance prediction in Aspergillus fumigatus
Source: J Clin Microbiol. 2025 Jul 1;63(8):e00291-25. doi: 10.1128/jcm.00291-25 (PMC12345187; doi:10.1128/jcm.00291-25)
Supplement: Supplemental material — Fig. S1, Tables S1 to S3, and bioinformatics protocol. [file jcm.00291-25-s0001.docx]

**SUPPLEMENTARY FILES**

**SUPPLEMENTARY FIGURES:**

**
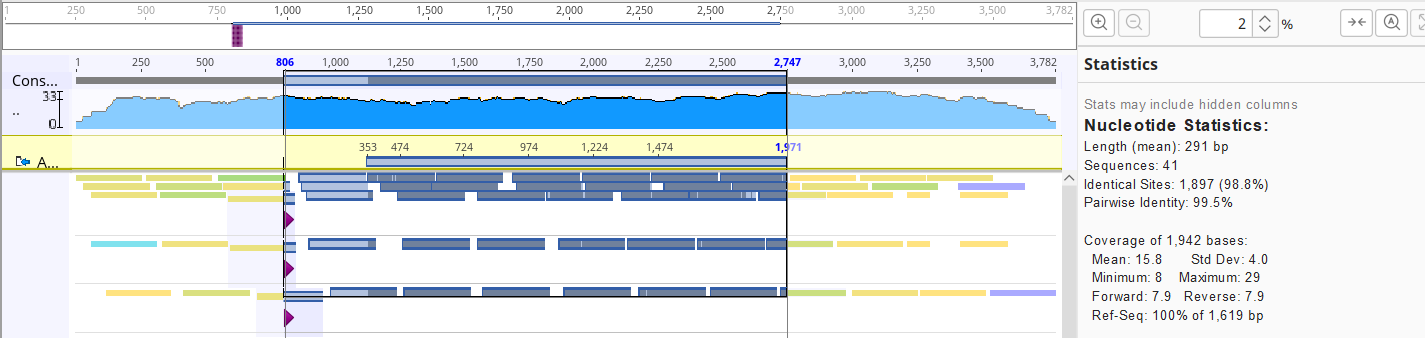
**


**
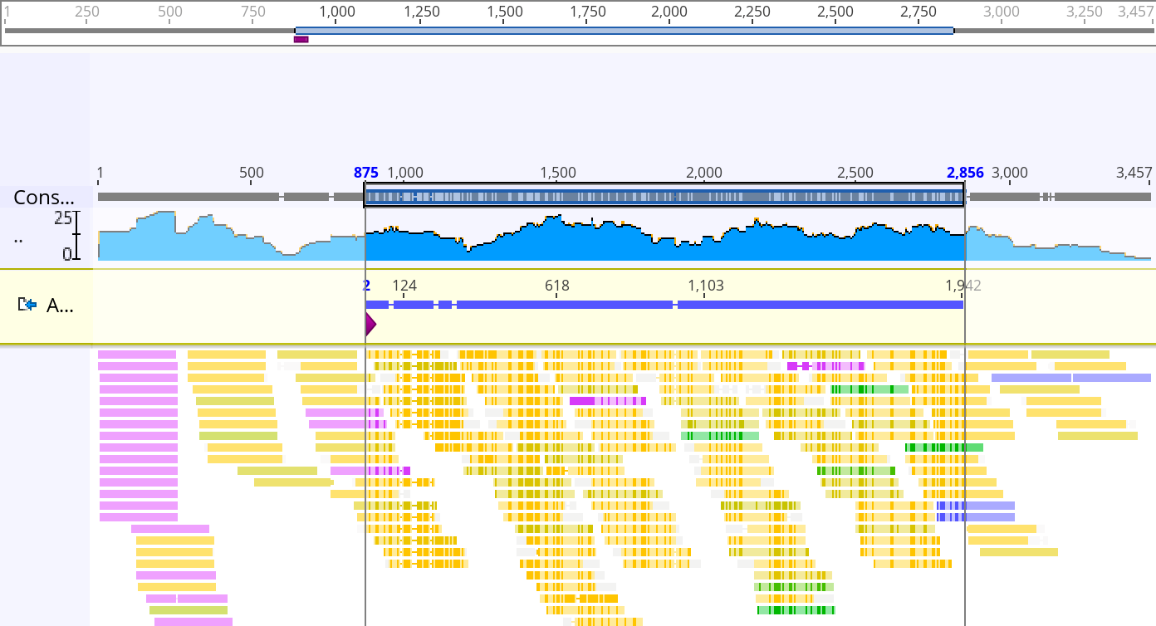
**

1. **
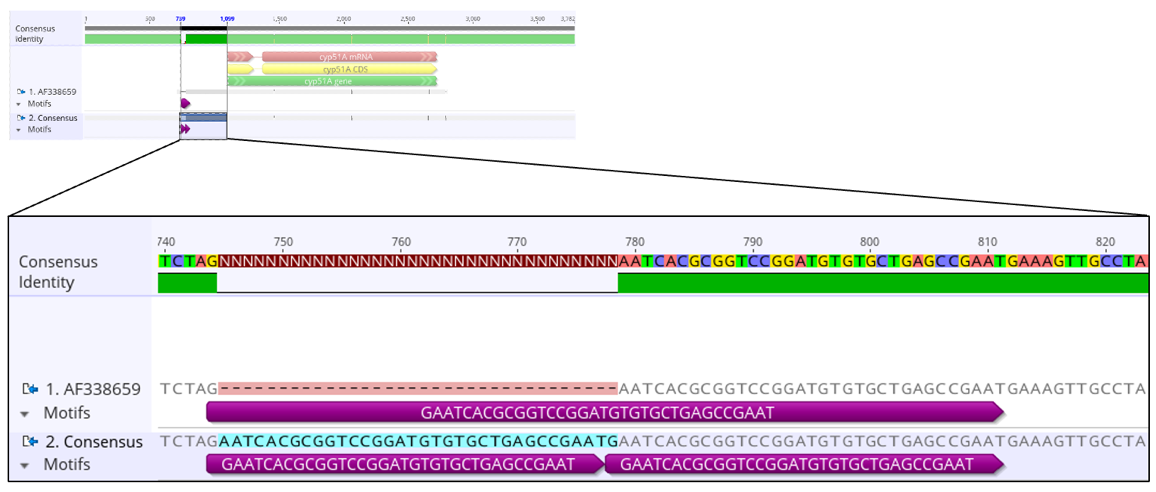
**

1. **
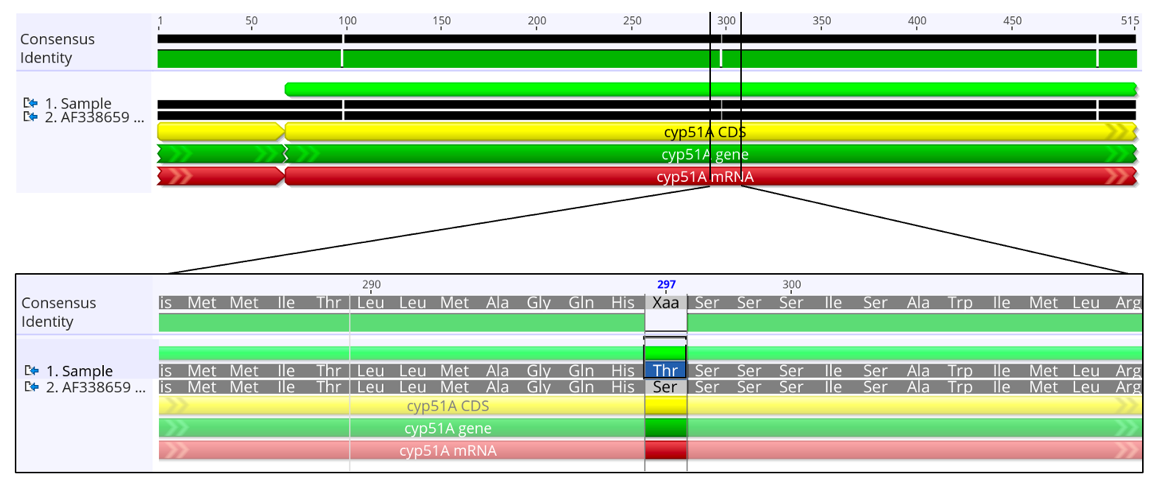
**

**Supplementary Figure 1. Bioinformatic analyses using Geneious Prime software. (A, Top Panel)** Mapped assembly using 1,941bp reference sequence (highlighted region) consisting of *cyp51A* CDS + 322bp upstream sequence (*cyp51A* promoter) (NCBI AF338659). Violet triangle marks position of tandem-repeat mutation. **(A, Bottom Panel)** Mapped assembly of non-*A. fumigatus* isolate (*A. lentulus*) demonstrating poor alignment to reference. **(B)** Nucleotide alignment to determine presence of tandem-repeat mutation. Zoomed inset on bottom panel: TR34 mutant (bottom) demonstrates 34bp tandem repeat mutation compared to wildtype reference AF338659 (top). **(C)** Protein alignment to determine presence of non-synonymous mutation. Zoomed inset on bottom panel: Amino acid on “Sample” sequence (top) denotes mutation compared to wildtype reference AF338659 (bottom).

**SUPPLEMENTARY TABLES**

| **Sample No.** | **Sample ID** | **Voriconazole μg/mL** | **Posaconazole μg/mL** | **Isavuconazole μg/mL** | **Itraconazole μg/mL** |
| --- | --- | --- | --- | --- | --- |
| **1** | **UCLA-001** | 0.5 | 0.06 | 0.5 | N/A |
| **2** | **UCLA-002** | 0.25 | <=0.03 | 0.5 | N/A |
| **3** | **UCLA-003** | 0.5 | 0.06 | 1 | N/A |
| **4** | **UCLA-004** | 1 | 0.25 | 2 | N/A |
| **5** | **UCLA-005** | >16 | 4 | >16 | >16 |
| **6** | **UCLA-006** | 0.25 | 0.06 | 0.5 | 0.25 |
| **7** | **UCLA-007** | 0.25 | 0.06 | 0.5 | N/A |
| **8** | **UCLA-008** | 0.5 | 0.06 | 0.5 | 0.25 |
| **9** | **UCLA-009** | 0.5 | 0.125 | 0.5 | 0.5 |
| **10** | **UCLA-010** | 0.5 | 0.125 | 0.5 | 1 |
| **11** | **UCLA-011** | 0.5 | 0.125 | 0.5 | 1 |
| **12** | **UCLA-012** | 0.25 | 0.06 | 0.5 | 0.125 |
| **13** | **UCLA-013** | 0.25 | 0.125 | 0.5 | 0.5 |
| **14** | **UCLA-014** | 0.5 | 0.5 | 0.5 | 1 |
| **15** | **UCLA-015** | 0.25 | <=0.03 | 0.5 | 0.25 |
| **16** | **UCLA-016** | 0.25 | 0.125 | 0.5 | 0.125 |
| **17** | **UCLA-017** | 0.25 | <=0.03 | 0.06 | 0.06 |
| **18** | **UCLA-018** | 0.5 | 0.06 | 0.5 | 0.5 |
| **19** | **UCLA-019** | >16 | 0.5 | >16 | 0.5 |
| **20** | **UCLA-020** | 0.5 | 0.06 | 1 | N/A |
| **21** | **UCLA-021** | 0.5 | 0.25 | 1 | N/A |
| **22** | **UCLA-022** | 0.5 | 0.25 | 0.5 | 1 |
| **23** | **UCLA-023** | 0.5 | 0.125 | 1 | N/A |
| **24** | **UCLA-024** | >16 | 0.5 | >16 | 1 |
| **25** | **UCLA-025** | 0.5 | 0.125 | 1 | 0.25 |
| **26** | **UCLA-026** | 1 | 0.125 | 0.5 | 0.25 |
| **27** | **UCLA-027** | 1 | 0.06 | 1 | N/A |
| **28** | **UCLA-028** | 0.5 | 0.06 | 1 | 0.5 |
| **29** | **UCLA-029** | 0.5 | 0.125 | 0.5 | 0.125 |
| **30** | **UCLA-030** | 0.5 | 0.06 | 1 | 0.5 |
| **31** | **AR-0731/ASP-01** | 4 | 0.5 (2) | 4 (>8) | >16 |
| **32** | **AR-0732/ASP-02** | 2 | 0.5 (2) | >16 | >16 |
| **33** | **AR-0733/ASP-03** | 4 (8) | 0.5 (2) | 4 (>8) | >16 |
| **34** | **AR-0734/ASP-04** | 8 | 0.5 (2) | 8 (>8) | >16 |
| **35** | **AR-0735/ASP-05** | 1 (2) | 0.5 (2) | 8 (>8) | >16 |
| **36** | **AR-0736/ASP-06** | 0.5 | 0.06 (0.12) | 0.5 (2) | 0.125 (0.5) |
| **37** | **AR-0737/ASP-07** | 0.125 (1) | 0.03 (0.25) | 0.125 (2) | 0.06 (0.5) |
| **38** | **AR-0738/ASP-08** | 1 (0.5) | 0.06 (0.12) | 0.5 (1) | 0.125 (0.5) |
| **39** | **AR-0739/ASP-09** | 0.25 (0.12) | 0.06 (0.03) | 0.25 | 0.25 |
| **40** | **AR-0740/ASP-10** | 0.5 (1) | 0.125 (0.25) | 1 (2) | 0.125 (0.5) |
| **41** | **UTHSA-DI20-081** | 4 | 1 | 4 | N/A |
| **42** | **UTHSA-DI20-082** | 2 | 0.5 | 2 | N/A |
| **43** | **UTHSA-DI20-084** | 2 | 1 | 4 | N/A |
| **44** | **UTHSA-DI20-086** | 2 | 0.25 | 1 | N/A |
| **45** | **UTHSA-DI20-091** | 4 | 0.5 | 4 | N/A |
| **46** | **UTHSA-DI20-096** | 2 | 0.25 | 2 | N/A |
| **47** | **UTHSA-DI20-098** | 2 | 8 | 2 | N/A |
| **48** | **UTHSA-DI20-100** | 2 | 0.5 | 4 | N/A |
| **49** | **UTHSA-DI20-102** | >16 | 1 | >16 | N/A |
| **50** | **UTHSA-DI20-103** | >16 | 0.25 | >16 | N/A |
| **51** | **UTHSA-DI20-106** | >16 | 0.5 | >16 | N/A |
| **52** | **UTHSA-DI20-108** | 2 | 0.125 | 0.5 | N/A |
| **53** | **UTHSA-DI20-112** | 4 | 2 | 8 | N/A |
| **54** | **UTHSA-DI20-113** | 2 | 0.25 | 4 | N/A |
| **55** | **UTHSA-DI20-115** | 2 | 2 | 4 | N/A |
| **56** | **UTHSA-DI20-128** | 4 | 1 | 8 | N/A |
| **57** | **UTHSA-DI20-132** | 4 | >16 | 8 | N/A |
| **58** | **UTHSA-DI20-134** | >16 | 2 | >16 | N/A |
| **59** | **UTHSA-DI20-180** | 1 | 1 | 2 | N/A |
| **60** | **UTHSA-DI21-184** | 1 | 1 | 2 | N/A |
| **61** | **UTHSA-DI21-187** | 0.25 | 1 | 2 | N/A |
| **62** | **UTHSA-DI21-188** | 0.25 | 1 | 0.5 | N/A |
| **63** | **UTHSA-DI21-192** | 0.25 | 1 | 0.5 | N/A |
| **64** | **UTHSA-DI21-193** | 0.5 | 1 | 0.5 | N/A |
| **65** | **UTHSA-DI24-307** | 0.5 | 0.125 | 1 | 0.125 |
| **66** | **UTHSA-DI24-308** | 0.5 | 0.125 | 1 | 0.5 |
| **67** | **UTHSA-DI24-309** | 0.5 | N/A | N/A | 0.125 |
| **68** | **UTHSA-DI24-310** | 0.25 | 0.06 | 0.5 | 0.5 |
| **69** | **UTHSA-DI24-311** | 0.5 | 0.125 | N/A | 0.25 |
| **70** | **UTHSA-DI24-312** | 0.5 | 0.125 | 1 | 0.125 |
| **71** | **UTHSA-DI24-313** | 0.5 | 0.125 | 1 | 0.125 |
| **72** | **UTHSA-DI24-314** | 0.5 | 0.06 | N/A | 0.125 |
| **73** | **UTHSA-DI24-315** | 0.25 | 0.06 | 0.5 | 0.125 |
| **74** | **UTHSA-DI24-316** | 0.5 | 0.06 | N/A | 0.125 |
| **75** | **UTHSA-DI24-317** | 0.5 | 0.06 | N/A | 0.125 |
| **76** | **UTHSA-DI24-318** | 0.25 | 2 | N/A | >16 |
| **77** | **UTHSA-DI24-319** | 0.25 | 0.125 | 0.25 | 0.5 |
| **78** | **UTHSA-DI24-320** | 0.25 | 0.125 | N/A | 0.5 |
| **79** | **UTHSA-DI24-321** | 8 | 0.5 | N/A | 0.5 |
| **80** | **UTHSA-DI24-322** | 0.5 | 0.125 | 1 | 0.125 |
| **81** | **UTHSA-DI24-323** | 0.5 | 0.125 | N/A | 0.125 |
| **82** | **UTHSA-DI24-325** | 0.5 | 0.125 | 1 | 0.125 |
| **83** | **UTHSA-DI24-326** | 0.25 | 0.06 | 0.5 | 0.25 |
| **84** | **UTHSA-DI24-327** | 0.5 | 0.25 | N/A | 0.25 |
| **85** | **UTHSA-DI24-328** | 0.25 | 0.125 | N/A | 0.5 |
| **86** | **UTHSA-DI24-329** | 0.5 | 0.06 | N/A | 0.25 |
| **87** | **UTHSA-DI24-330** | 0.5 | 0.125 | 1 | 0.125 |
| **88** | **UTHSA-DI24-331** | 0.5 | 0.125 | N/A | 0.125 |
| **89** | **UTHSA-DI24-332** | 0.5 | 0.25 | 1 | 0.25 |
| **90** | **UTHSA-DI24-333** | 0.5 | 0.125 | N/A | 0.25 |
| **91** | **UTHSA-DI24-334** | 0.25 | 0.06 | N/A | 0.25 |
| **92** | **UTHSA-DI24-335** | 0.5 | 0.06 | N/A | 0.125 |
| **93** | **UTHSA-DI24-336** | 1 | 0.125 | N/A | 0.25 |
| **94** | **UTHSA-DI24-337** | 0.5 | 0.125 | N/A | 0.25 |
| **95** | **UTHSA-DI24-338** | 0.5 | 0.25 | 1 | 0.5 |
| **96** | **UTHSA-DI24-339** | 0.5 | 0.125 | 1 | 0.25 |
| **97** | **UTHSA-DI24-340** | 0.5 | 0.125 | 0.5 | 0.25 |
| **98** | **UTHSA-DI24-341** | 0.5 | 0.06 | 0.5 | 0.125 |
| **99** | **UTHSA-DI24-342** | 0.5 | 0.06 | 0.5 | 0.125 |
| **100** | **UTHSA-DI24-343** | 0.25 | 0.06 | N/A | 0.125 |
| **101** | **UTHSA-DI24-344** | 0.25 | 0.06 | N/A | 0.125 |
| **102** | **UTHSA-DI24-345** | 0.25 | 0.06 | N/A | 0.125 |
| **103** | **UTHSA-DI24-346** | 0.5 | 0.06 | N/A | 0.5 |
| **104** | **UTHSA-DI24-347** | 0.5 | 0.06 | N/A | 0.125 |
| **105** | **UTHSA-DI24-348** | 0.25 | 0.06 | 0.5 | 0.25 |
| **106** | **UTHSA-DI24-349** | 0.5 | 0.125 | N/A | 0.125 |
| **107** | **UTHSA-DI24-350** | 0.5 | 0.25 | N/A | 0.125 |
| **108** | **UTHSA-DI24-351** | 0.25 | 0.5 | N/A | 0.06 |
| **109** | ***UTHSA-DI24-324*** | *1* | *0.25* | *0.5* | *0.06* |

**Supplementary Table 1. Phenotypic Antifungal Susceptibility Testing (AFST).** AFST was performed at the UT Health at San Antonio FTL by broth microdilution (BMD) according to current CLSI guidelines. Values in parentheses are MICs reported by the CDC AR Bank. Isolate identified as *A. lentulus* by WGS analysis is marked in italics. N/A represent MICs not available.

| **Mutation/ Polymorphism** | **Number of Identified Mutations/ Polymorphisms** | **Percent Frequency of Mutation/Polymorphism in All Isolates** |
| --- | --- | --- |
| **A9T** | 1 | 2.86 |
| **M220I** | 1 | 2.86 |
| **M220K** | 1 | 2.86 |
| **M220R** | 1 | 2.86 |
| **N248K** | 1 | 2.86 |
| **N248T** | 1 | 2.86 |
| **D255E** | 1 | 2.86 |
| **I309V** | 1 | 2.86 |
| **N512I** | 1 | 2.86 |
| **G54R** | 2 | 5.71 |
| **P216L** | 2 | 5.71 |
| **I242V** | 2 | 5.71 |
| **S297T** | 2 | 5.71 |
| **F495I** | 2 | 5.71 |
| **F46Y** | 3 | 8.57 |
| **M172V** | 3 | 8.57 |
| **E427K** | 3 | 8.57 |
| **TR46** | 4 | 11.43 |
| **G448S** | 4 | 11.43 |
| **T289A** | 6 | 17.14 |
| **Y121F** | 7 | 20.00 |
| **TR34** | 9 | 25.71 |
| **L98H** | 9 | 25.71 |
| **Total No. of Isolates** | **35** | **32%** |

**Supplementary Table 2. Frequency of each mutation/polymorphism among the UCLA-sequenced isolates.** The frequency of each mutation or polymorphism was calculated as the percent of isolates with each detected mutation among all non-wildtype isolates. The total frequency of mutation/polymorphism is the percent of isolates with any detected mutation among all UCLA-sequenced isolates.

| **Source** | | | **Voriconazole** | **Posaconazole** | **Itraconazole** | **Isavuconazole** |
| --- | --- | --- | --- | --- | --- | --- |
| **UCLA-sequenced Sample Set** | **Sequencing Development and Validation Subset** | **CDC AR Bank** | 10 | 10 | 10 | 10 |
|  |  | **UT Health San Antonio** | 24 | 24 | 0 | 24 |
|  | **Clinical Subset** | **UCLA Clinical Microbiology Lab** | 30 | 30 | 30 | 21 |
|  |  | **UT Health San Antonio** | 44 | 43 | 44 | 18 |
| **Literature** | | **14 Peer-reviewed Studies** | 392 | 633 | 414 | 72 |
| **Total** | | | **500** | **740** | **498** | **145** |

**Supplementary Table 3. Number of isolates included in comprehensive data set.** The count (n) of isolates with corresponding phenotypic-genotypic data for each triazole drug utilized in the comprehensive dataset.

**Step-by-step procedure on Triazole Resistance Assay bioinformatic protocol using Geneious Prime software version 11.**

**Creating References**:

1. Create a new folder for references using the “Add (+)” drop-down menu at the top of the screen and selecting “New Folder.” Label the new folder, “References.”
2. Download the reference sequence AF338659:
3. On the lefthand panel, locate the “NCBI” folder, and click the “Nucleotide” subfolder to open the search window.


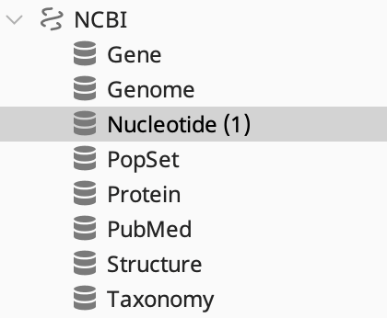


1. Using the search window, search for the sequence AF338659, “*Aspergillus fumigatus* 14-alpha sterol demethylase (cyp51A) gene, complete cds”
2. Drag and drop the sequence onto the “References” folder to save.
3. Create the following references for analysis:
4. Mapping/Assembly Reference:
   1. Single-click on the downloaded reference sequence to view the sequence.


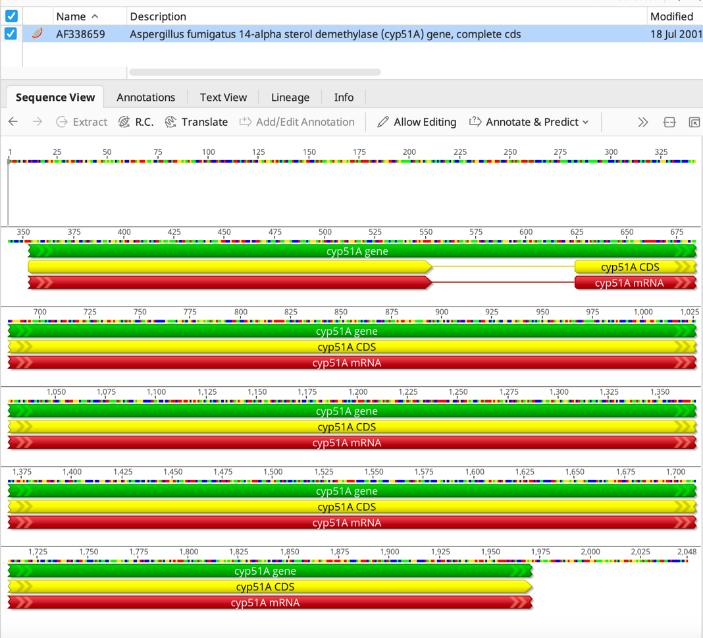


- 1. Under the “Sequence View” tab, click the “Annotate & Predict” drop-down menu, and click “Find Motifs”
  2. In the new window, insert the following 34-base nucleotide sequence in the “Sequence or PROSITE motif:” text box, then click “OK:” GAATCACGCGGTCCGGATGTGTGCTGAGCCGAAT


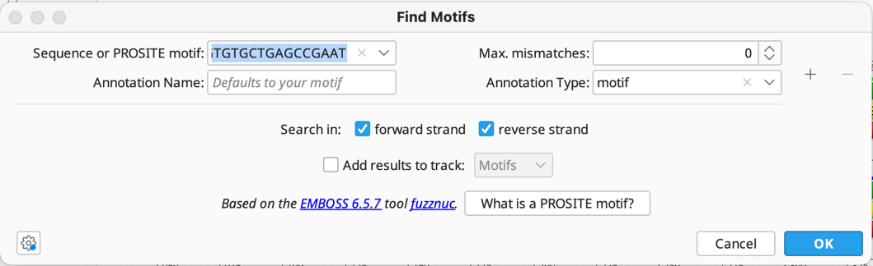


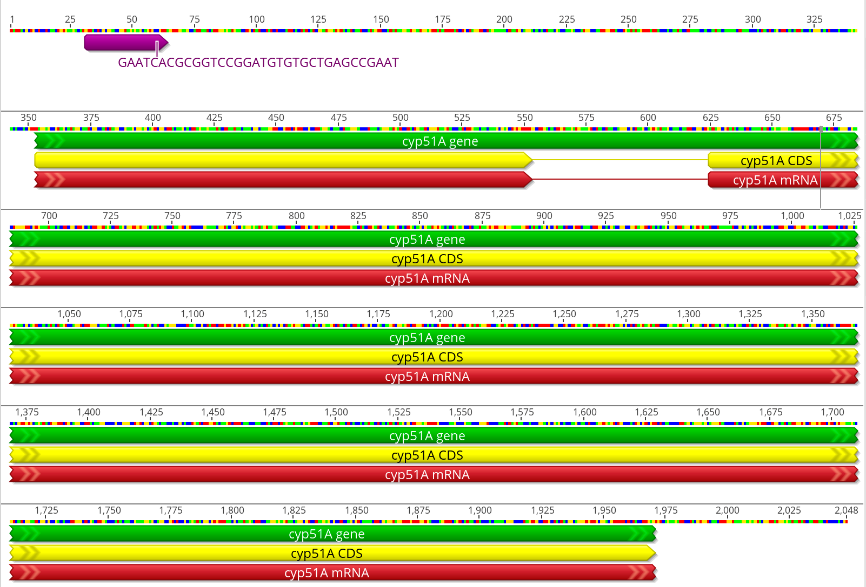


The motif will be marked in violet in the reference sequence, upstream of the coding sequence (CDS). Click the “Save” button next to the “Annotate & Predict” drop-down menu.

- 1. Starting at the first base of the motif (G), select the nucleotide sequence up to the final nucleotide of the CDS (A). Right click on the selected sequence and click “Copy sequence name and bases.”
  2. Create a new sequence by using the “Add (+)” drop-down menu at the top of the screen and selecting “New Sequence.”
  3. In the new window, paste the copied nucleotide sequence in the text box.
     1. Under Name, type “AF338659 – Assembly Reference,” and under Description, type “TR34 Motif + CD4.” Under “Type,” select “Nucleotide.” Click OK.
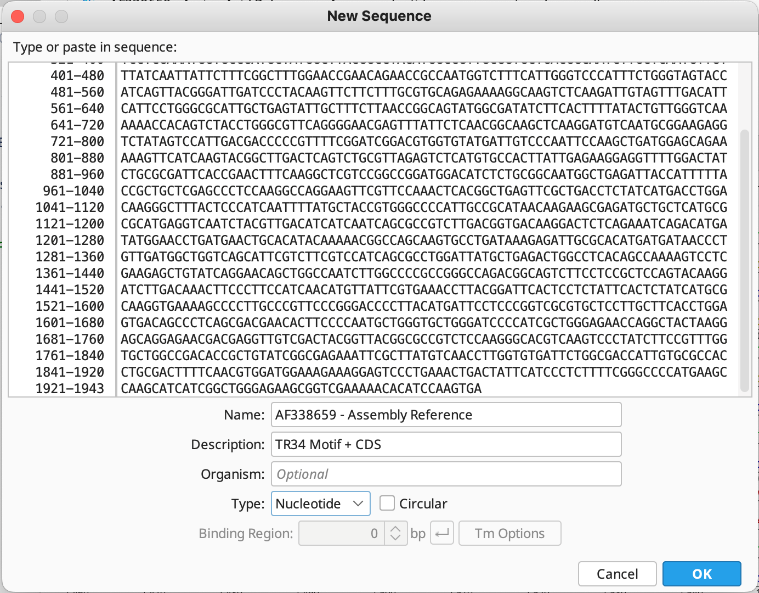


1. Nucleotide Alignment Reference:
   1. Use the downloaded AF338659 sequence without modifications.
2. Amino Acid Alignment Reference:
   1. Single-click on the downloaded reference sequence to view the sequence.
   2. Concatenate the two coding domains of the CDS (yellow) by individually copying and pasting the nucleotide sequences.
      1. Select and copy the first domain, then create a new sequence as above.
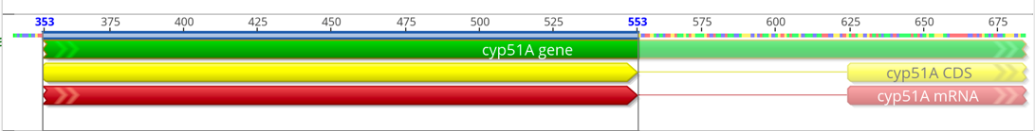

         1. Under Name, type “AF338659 – CDS Reference.” Click OK.
      2. Select and copy the second domain.
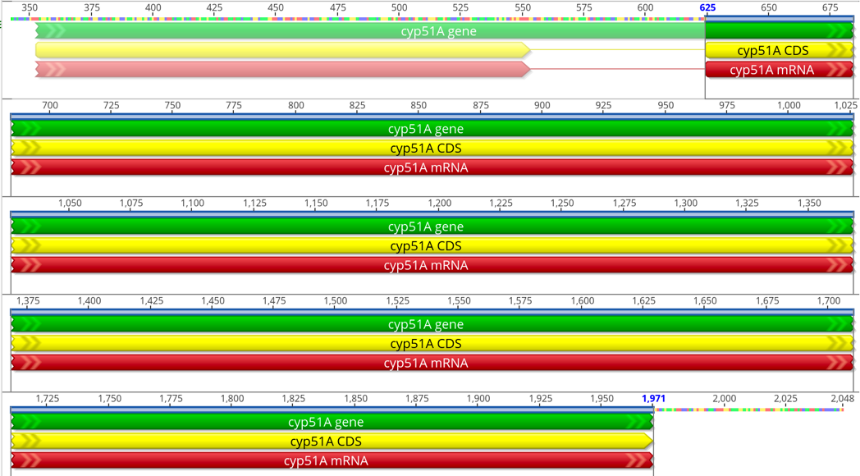

      3. Open the new “AF338659 – CDS Reference,” and click “Allow Editing” under the “Sequence View” tab.
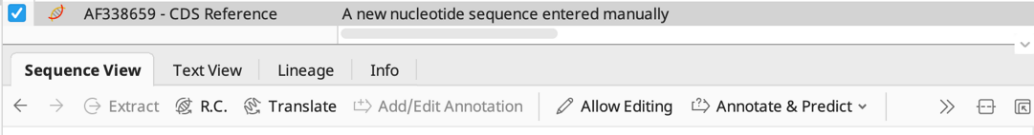

      4. Paste the nucleotide sequence of second domain at the end. Click “Save.”


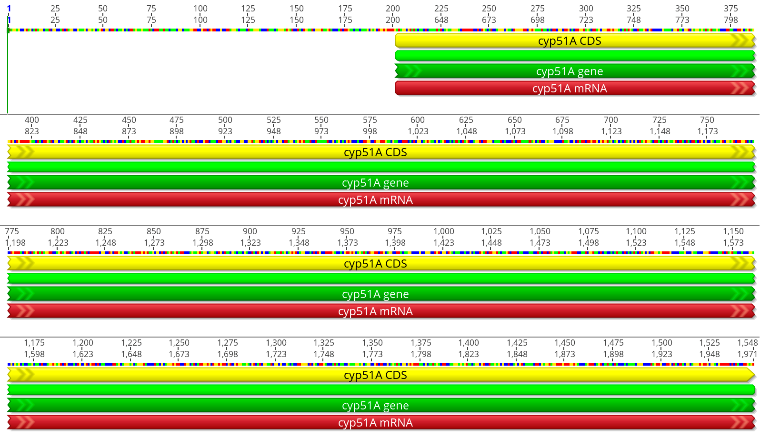


- - 1. Under the “Sequence View” tab, click “Translate.”
       1. Under “Extraction name,” change the default label to “AF338659 – Amino Acid Reference”

**Performing the analysis**:

1. Importing files:
2. Create a new folder for analysis using the “Add (+)” drop-down menu at the top of the screen and selecting “New Folder.” Label the new folder, “Azole Resistance Analyses.”
   1. Create a subfolder for each run labeled with the run file name (e.g. WGS_2024_08_01_CF-426632798)
3. Import paired reads using the “Add (+)” drop-down menu and selecting “Import Files.” Locate and select the paired reads and click Open.

NOTE: Make sure the intended project folder is clicked to ensure import into the appropriate folder (e.g. “WGS_2024_08_01_CF-426632798”).

1. Performing resistance analysis:
2. Assemble paired reads to “AF338659 - Assembly Reference.”

NOTE: Assembly must be performed individually for all files (i.e. cannot be batched).

- 1. Single-click to select the file, then using the “Align/Assemble” drop-down menu at the top of the screen, select “Map to Reference(s)…”
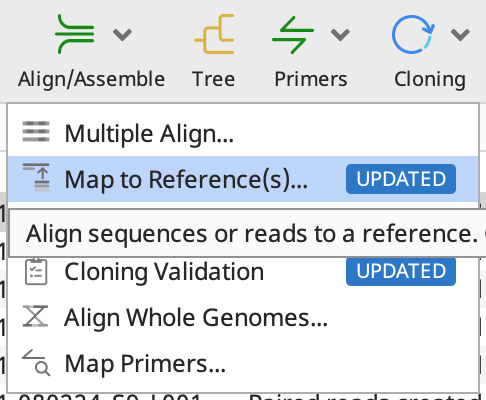

  2. A new window will appear.
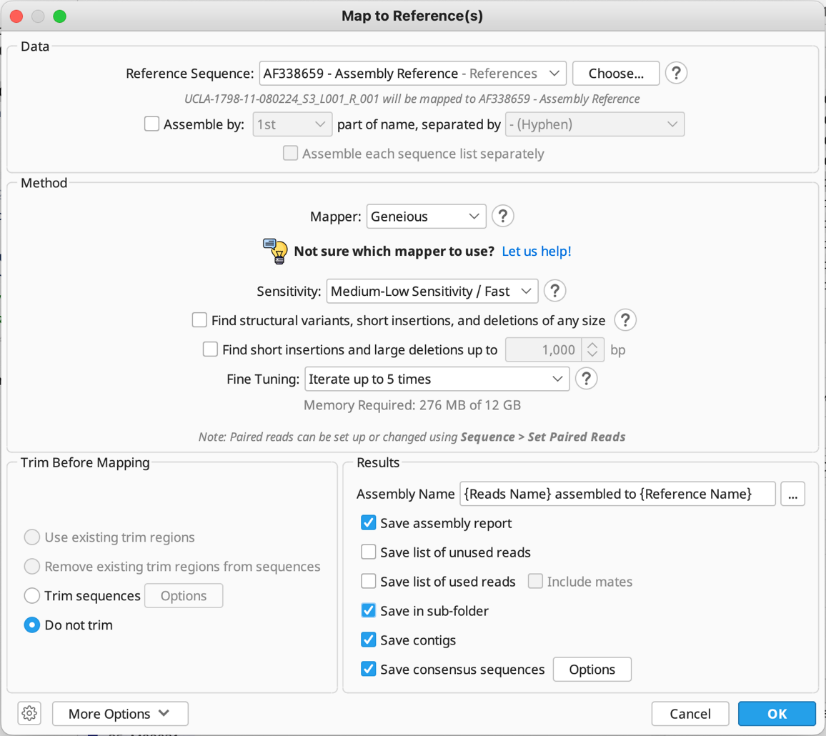

     1. In the “Data” panel, select the appropriate reference sequence by clicking “Choose” and locating “AF338659 - Assembly Reference.” Click “Select.”
     2. In the “Method” panel, use the “Geneious” Mapper, with “Medium-Low Sensitivity/Fast” option. Make sure all other options are unclicked.
     3. In the “Trim Before Mapping” panel, click “Do not trim.”
     4. In the “Results” panel, click the following options (and make sure all others are unclicked. Click OK.
        1. Save assembly report
        2. Save in sub-folder
        3. Save contigs
        4. Save consensus sequences
     5. A new subfolder will be created with the following files:
        1. Assembly Report
        2. Consensus
        3. Contig

1. Record quality metrics
   1. Assembled Reads:
      1. In the subfolder produced after assembly, single-click the “Assembly Report” file to view. Record the assembled reads and total reads.
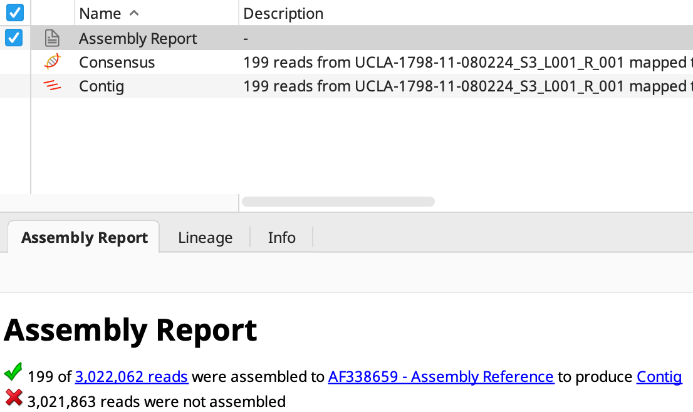

   2. Coverage:
      1. Single-click the “Contig” file to view. In the “Contig View” tab, using the “Annotate & Predict” drop-down menu, select “Find Motifs.” Type the following nucleotide sequence in the “Sequence of PROSITE motif:” text box: GAATCACGCGGTCCGGATGTGTGCTGAGCCGAAT. Click OK, then click the “Save” button next to the “Annotate & Predict” drop-down menu.

The motif will be marked in violet.

- - 1. Starting from the first nucleotide in the marked motif (G), select the contig sequence until the end of the assembly reference (marked by purple bar) (A). In cases where tandem-repeats are identified, start with the upstream-most (first) motif.
    2. In the righthand-most panel, click the “%” tab to view assembly statistics. Record Mean and Minimum coverage.


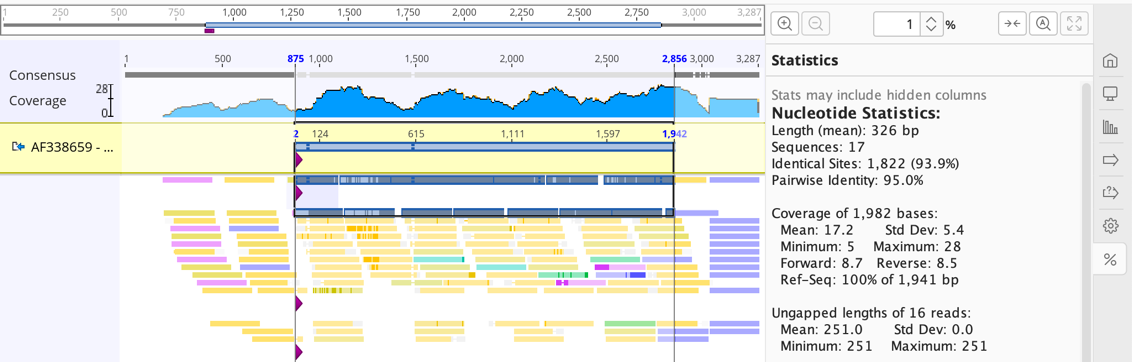


1. Pairwise nucleotide alignment to identify promoter-region tandem-repeat mutations.
   1. Copy “AF338659” file from the “References” folder and paste onto sample subfolder.
   2. Select the AF338659 and Consensus files by clicking the check boxes adjacent to the file names.


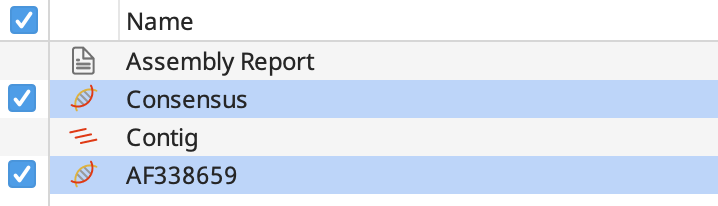


- 1. Using the Align/Assemble drop-down menu at the top of the screen, select “Pairwise align…”
  2. In the new window, use all default options then click OK.
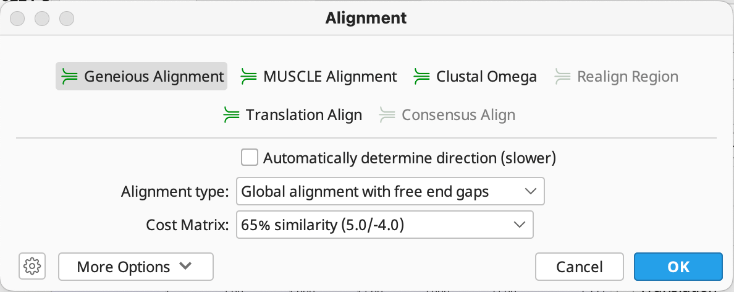

  3. Single-click the “Nucleotide alignment” file to view.
  4. In the “Alignment View” tab, using the “Annotate & Predict” drop-down menu, select “Find Motifs.” Type the following nucleotide sequence in the “Sequence of PROSITE motif:” text box: GAATCACGCGGTCCGGATGTGTGCTGAGCCGAAT. Click OK, then click the “Save” button next to the “Annotate & Predict” drop-down menu.
  5. Locate the motif marked in violet and determine the presence of a tandem-repeat mutation.
     1. 34-base tandem-repeat (TR34) will be marked as two immediately consecutive motifs
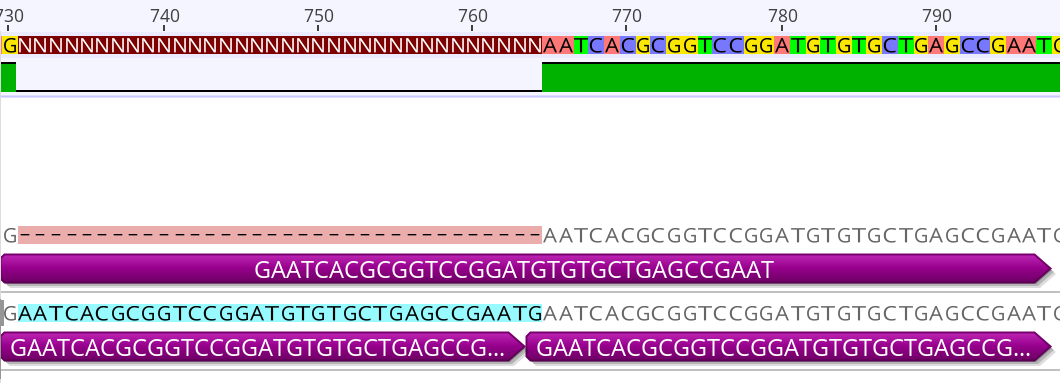

     2. 46-base tandem-repeat (TR46) will be marked by two motifs followed by a an additional 12-base sequence (GAAAGTTGTCTA)
     3.
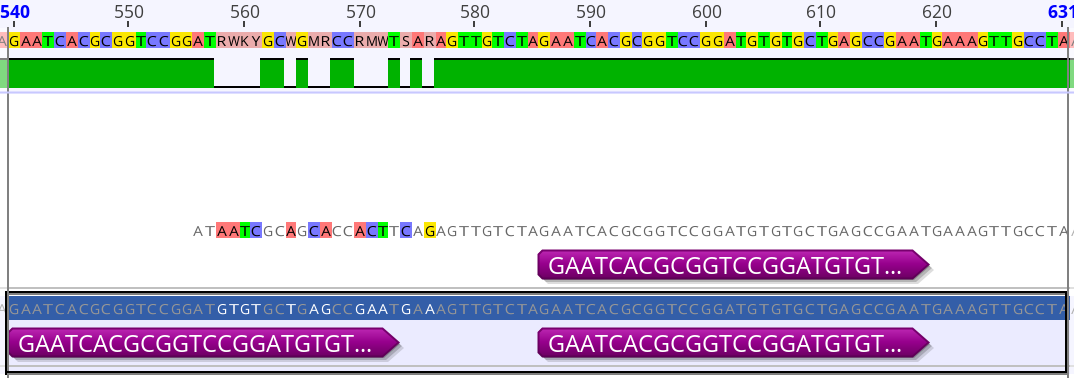

     4. Record presence of mutation if identified, and the corresponding coverage metrics as above.
        1. Single-click the “Contig” file and select the nucleotide sequence of the tandem-repeat mutation.

NOTE: Be sure to include the additional 12-base sequence for TR46 mutations.

- - - 1. Click the “%” tab and record Mean and Minimum coverage.

1. Pairwise amino acid alignment to identify non-synonymous coding sequence mutations.
2. Copy “AF338659 – Amino Acid Reference” file from the “References” folder and paste onto sample subfolder.
3. Single-click the “Nucleotide Alignment” file to view. Copy and concatenate the two coding domains of the CDS as above, labeling the new sequence as “UCLA_XXXX CDS.”

NOTE: Make sure to hover above the “Consensus” sequence (i.e. NOT “Consensus Identity” or “AF338659”). Click Save.

1. Translate the CDS by click the “Translate” button. In the new window, use the default label on “Extraction name,” and make sure box option boxes are checked. Click OK.


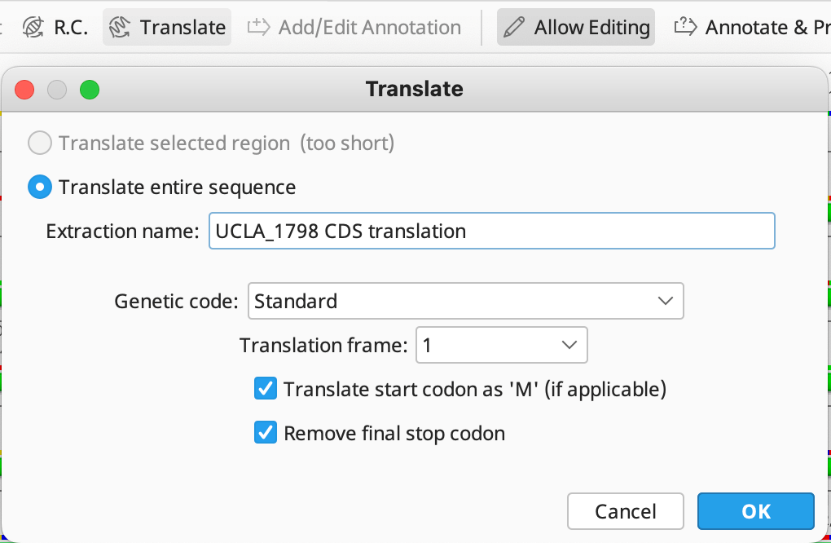


1. Select the “AF338659 – Amino Acid Reference” and “UCLA_XXXX CDS Translation” files by clicking the check boxes adjacent to the file names.
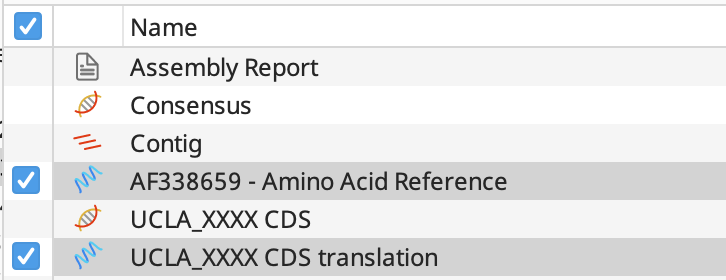

2. Using the Align/Assemble drop-down menu at the top of the screen, select “Pairwise align…”
3. In the new window, use the default “Geneious Alignment” option, then click OK.
4. Determine the presence of non-synonymous mutation(s) marked by gaps in alignment in the “Consensus Identity.”
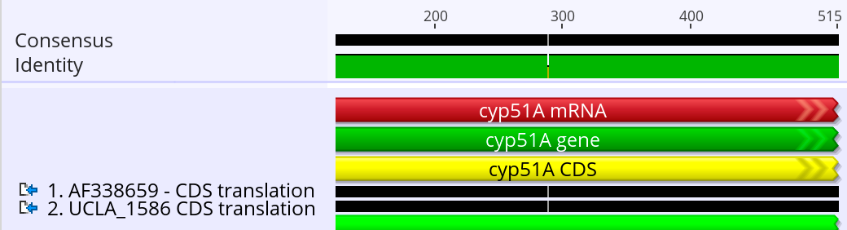

5. Record presence of mutation(s) if identified, and the corresponding coverage metrics as above.
   - - 1. Single-click the “Contig” file and select mutant nucleotide corresponding to the non-synonymous mutation, which can be identified by the black lines in the “Consensus” sequence.
       2. Click the “%” tab and record Mean and Minimum coverage, as above.
